# Noncoding function of super enhancer derived *Cpox* pre-mRNA in modulating neighbouring gene expression and chromatin interactions

**DOI:** 10.1080/15476286.2025.2475421

**Published:** 2025-03-06

**Authors:** Bingning Xie, Ann Dean

**Affiliations:** Laboratory of Cellular and Developmental Biology, National Institute of Diabetes and Digestive and Kidney Diseases, National Institutes of Health, Bethesda, MD, USA

**Keywords:** Chromatin structure, chromatin regulation, super enhancer, TAD boundary, Inter-gene interaction, gene silencing, oncogene

## Abstract

Super enhancers are important regulators of gene expression that often overlap with protein-coding genes. However, it is unclear whether the overlapping protein-coding genes and the RNA derived from them contribute to enhancer activity. Using an erythroid-specific super enhancer that overlaps the *Cpox* gene as a model, *Cpox* pre-mRNA is found to have a non-coding function in regulating neighbouring protein-coding genes, eRNA expression and TAD interactions. Depletion of *Cpox* pre-mRNA leads to accumulation of H3K27me3 and release of p300 from the *Cpox* locus, activating an intra-TAD enhancer and gene expression. Additionally, a head-to-tail interaction between the TAD boundary genes *Cpox* and *Dcbld2* is identified, facilitated by a novel type of repressive loop anchored by p300 and PRC2/H3K27me3. These results uncover a regulatory role for pre-mRNA transcribed within a super enhancer context and provide insight into head-to-tail inter-gene interaction in the regulation of gene expression and oncogene activation.

## Introduction

Super enhancers are clusters of transcriptional enhancers that are collectively bound by groups of transcription factors and mediator, and play an important role in regulating cell identity genes [1]. Some super enhancers overlap with protein-coding genes [1,2], but it is unclear whether these genes can function as enhancers themselves or whether the RNA transcribed from them plays any role as an eRNA.

Controlling gene expression is a complex process that involves not only the direct interaction between an enhancer and its target gene but also indirect influences from the expression status of other genes. For instance, the enhancer release and re-targeting (ERR) model proposes that repressing gene expression through transcription start site (TSS) deletion or dCas9-KRAB-based transcriptional repression can release its partner enhancer, which can then retarget to other neighbouring genes, activating their expression [3]. The involvement of pre- and mature mRNA in this process and the mechanism by which enhancer activation occurs in response to pre- and mature mRNA loss are currently unknown.

Loss of transcription by TSS deletion results in histone deacetylation, which mediates Ras-induced gene silencing and leads to accumulation of H3K27me3 in the gene body [4]. PRC2, which deposits H3K27me3, can interact with both RNA and chromatin, in a competitive fashion [5]. Nascent RNA–PRC2 interaction can inhibit PRC2 function in situ, as demonstrated by the long non-coding RNA LEVER, which is transcribed from upstream of the β-globin cluster and interacts with PRC2 in its nascent form. CRISPR-Cas9-mediated knock-out or CRISPRi-based transcriptional inhibition of LEVER triggers PRC2 translocation to chromatin, leading to altered interaction between the LEVER gene locus and its target sites and, subsequently, activation of neighbouring ε-globin gene expression [6]. However, it remains unclear whether the RNA molecule itself is involved in this process. Thus, currently, there is no direct evidence to support the hypothesis that H3K27me3 accumulation following TSS deletion is caused by PRC2 moving from RNA to chromatin.

The genome in the nucleus is organized into different layers of higher architecture, ranging from nucleosome to chromatin looping, to topologically associated domains (TADs) and A/B compartments [7,8]. Some TAD boundaries overlap with protein-coding genes or non-coding genes [9–12]. The non-coding RNA transcribed from a TAD boundary (bRNA) has been reported to strengthen TAD insulation by recruiting CTCF to the TAD boundary [9]. R loops, which are DNA:RNA hybrids, form as natural byproducts of transcription and accumulate around transcription start and termination sites [13]. They have been found to facilitate and stabilize chromatin loops and TADs [14–17]. However, whether pre- and mature mRNA transcribed from TAD boundaries also has a regulatory role in TAD structure is currently unclear. Actively transcribed genes located at TAD boundaries have been reported to function as boundary elements. For example, the transcribed α2-globin gene, but not a nearby CTCF site, was the anchor for the TAD boundary in the α-globin locus [11]. Nevertheless, it is not known whether genes located at the two boundaries of a TAD regulate each other.

In this study, we demonstrate that *Cpox*, which overlaps with a super enhancer located at a TAD boundary, transcribes a pre-mRNA that has a non-coding function in regulating enhancer and neighbouring gene expression as well as intra-TAD interactions. Intra-TAD protein coding gene *St3gal6* and enhancer *CpoxeRNA* interact with the gene body region of *Cpox*. The two TAD anchor genes, *Cpox* and *Dcbld2*, are organized in a head-to-tail inter-gene conformation. A novel type of repressive loop, established by a p300 only peak in the *Cpox* intron and H3K27me3 in the promoter of *Dcbld2*, represses *Dcbld2* gene expression. Loss of *Cpox* pre-mRNA triggers H3K27me3 accumulation on chromatin, which alters these chromatin interactions and releases transcription activators from the *Cpox* locus, resulting in the activation of the enhancer and protein coding genes *St3gal6* and *Dcbld2* within the TAD. This, in turn, leads to increased transcriptional R-loops and strengthens TAD corner loop interaction. Analysis of cancer mutations shows that the head-to-tail TAD boundary gene conformation is a potential mechanism to harness oncogene activation.

## Materials & methods

### Cell culture

MEL cells were cultured in Dulbecco’s modified Eagle’s medium supplemented with 10% foetal bovine serum in a humidified incubator with 5% CO_2_. Differentiation of MEL cells was induced by 2% dimethylsulfoxide for 5 days.

### Knockdown experiments: shRNA and ASO

shRNA knockdown experiments were performed by electroporation of shRNA (Sigma SuperScript™ shRNA (shCPOX-4 and shCPOX-5) and custom designed shRNA targeting exon–exon junction and exon 2 by using programs from Invivogen and VectorBuilder) into MEL cells with BTXpress High-Performance Electroporation Kit & Solution (BTXpress, catalogue no. 89130–538) and Biorad instrument. Cells were transferred to DMEM medium containing 20% FBS immediately after electroporation, incubated at 37°C for 2 days, and then cultured in DMEM medium with 10% FBS and 2 μg/ml puromycin (Thermofisher, catalogue no. A1113803) for 3 days. For shRNA knockdown in 293T cells, two shRNAs for each gene (except one shRNA for *PSMD3*) were transiently transfected to 293T cells with lipofectamine 2000 (ThermoFisher, Catalogue no. 11668019), selected with 2 μg/ml puromycin, and then collected for RNA isolation.

The ASO knock-down experiment was performed by electroporation of LNA (QIAGEN) into MEL cells with BTXpress High-Performance Electroporation Kit & Solution (BTXpress, catalogue no. 89130–538). Cells were transferred to a DMEM medium containing 20% FBS immediately after electroporation and incubated at 37°C for 2 days, then harvested for RNA isolation.

### CRISPR-Cas9 knock out

sgRNAs were designed by using CRISPick (Broad institute, MIT), cloned into pSpCas9(BB)-2A-Puro (PX459) V2.0 (pSpCas9(BB)-2A-Puro (PX459) V2.0 was a gift from Feng Zhang (Addgene plasmid # 62988; http://n2t.net/addgene:62988; RRID: Addgene_62988)) according to the protocol from Ran F et al. [18] For *Cpox* intron 5 TFBS deletion, sgRNA plasmids were electroporated into MEL cells with the BTXpress High-Performance Electroporation Kit & Solution (BTXpress, catalogue no. 89130–538). Cells were transferred immediately into DMEM medium with 20% FBS and cultured for 1 day, then treated with 4 μg/ml puromycin for 12 hours, washed and cultured in puromycin-free DMEM with 10% FBS. *Cpox* TSS deletion was performed with the same procedure except with continuous cultured in medium with 2 μg/ml puromycin to select permanent integrated cells. Selection of single colonies by serial dilution culture was performed in 96 well plates. Genotyping of single colonies was performed after extraction of DNA with QuickExtract DNA Extraction Solution (Biosearch Technologies, catalogue no. 76081–766) and PCR amplification with Q5® High-Fidelity 2X Master Mix (NEB, catalogue no. M0492S). PCR products were confirmed by Sanger sequencing (Genewiz).

### ORF overexpression experiment

*Cpox* ORF (pcDNA 3.1(+)-CPOX-ORF mutant) with mutated shRNA binding sites for both shRNA 4 and shRNA 5 were synthesized by GenScript. pcDNA 3.1(+)-mCherry was cloned from pHR_TRE3G-p300-dCas9-P2A-mCherry plasmid by PCR amplifying mCherry with primers containing NheI and KnpI restriction sites and inserting into pcDNA 3.1(+) backbone. 10 μg plasmid was used for electroporation, and subsequently 2 μg RNA were used from qRT-PCR experiment.

### Flow cytometry assays

Flow cytometry experiments were conducted on the FACS BD LSRFortessa machine (BD Biosciences). Cells were immunostained with PE Rat Anti-Mouse CD71 (BD Biosciences, Catalogue #: 553267), allophycocyanin-conjugated anti-TER119 (BD Biosciences, Catalogue #: 557909) antibodies, and 5 μg/mL Hoechst 33,342 (Thermo Fisher, Catalogue #: 62249) [19,20].

### Nascent RNA

Nascent RNA was detected by EU labelling experiments. Experiments were done according to the protocol of Click-iT Nascent RNA Capture kit (Thermo Fisher, Catalogue no. C10365). Briefly, cells were pulsed with 0.5 mm EU for 1 hour, total RNA was isolated, and 5 μg EU labelled RNA was used in a copper catalysed click reaction with azide-modified biotin. Then, 1 μg biotinylated RNA was captured on streptavidin magnetic beads and cDNA synthesis was performed with pull-downed material directly on the beads using the Superscript VILO cDNA synthesis kit (Thermofisher, Catalogue no. 11754050) followed by an analysis with qRT-PCR. See Supplementary Data for primers.

### RT-qPCR

**Figure 1.**
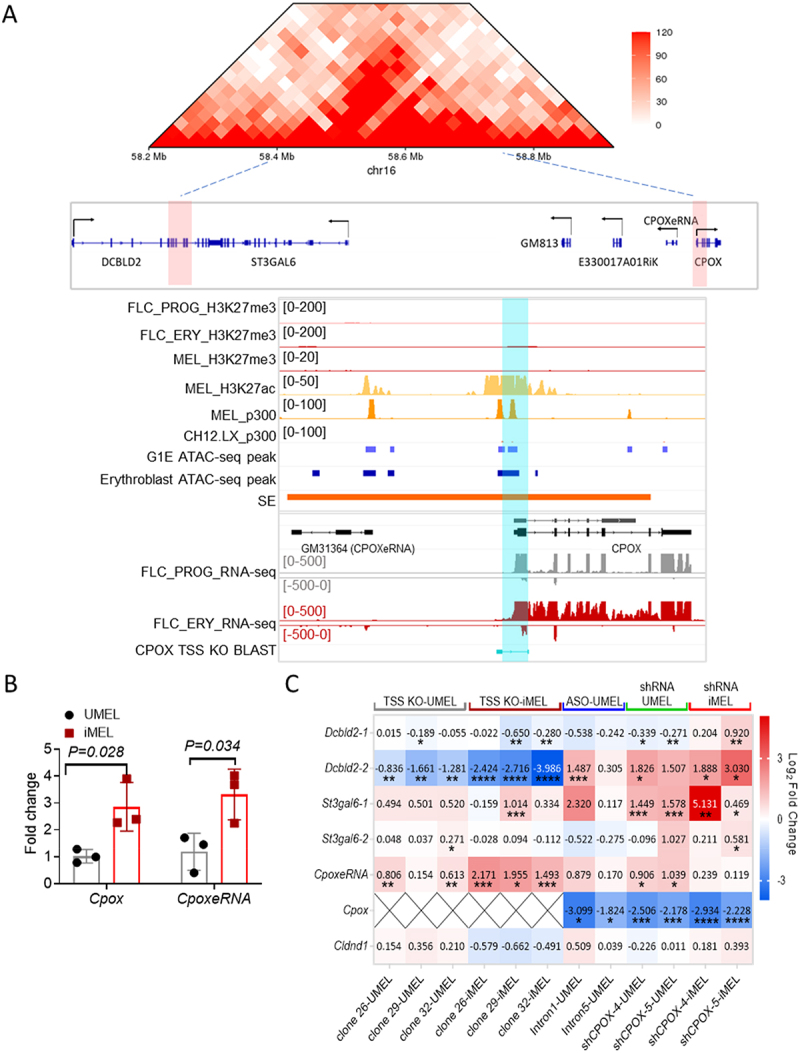
*Cpox* KD activates partner enhancer and neighbouring genes. A. 25kb resolution HiC map of the *Cpox* TAD from E14.5 foetal liver cells (upper panel). Genes located in this TAD are shown below. TAD boundaries are highlighted in red, black arrows represent transcription direction. IGV tracks show the super enhancer overlap with *Cpox*, H3K27me3 ChIP-seq and RNA-seq data from progenitor (FLC_PROG) and differentiated mouse foetal liver cells (FLC_ERY) [21], ENCODE atac-seq peaks for G1E cells and mouse erythroblast cells, ENCODE histone ChIP-seq for MEL cells and CH12.LX cells. *Cpox* TSS deleted region is highlighted in blue. B. qRT-pcr result shows *Cpox* and *CpoxeRNA* expression level in undifferentiated (UMEL) and differentiated (iMEL) MEL cells. Three biological replicates. Data are mean ± s.D., unpaired two-tailed t-test. C. Heatmap shows the qRT-pcr result of eRNA and gene expression levels in *Cpox* TSS deletion undifferentiated and differentiated MEL cells (independent clones 26, 29, and 32 are shown), *Cpox* knockdown with ASO in undifferentiated MEL cells, and *Cpox* knockdown with shRNA in undifferentiated and differentiated MEL cells. Value in each cell represents log2 fold change relative to corresponding control samples, data normalize with ACT. *: *p* ≤ 0.05; **: *p* ≤ 0.01, ***: *p* ≤ 0.001, ****: *p* ≤ 0.0001. Two biological replicates each except for iMEL-tss KO and shRNA-iMEL which have three biological replicates.

**Figure 2.**
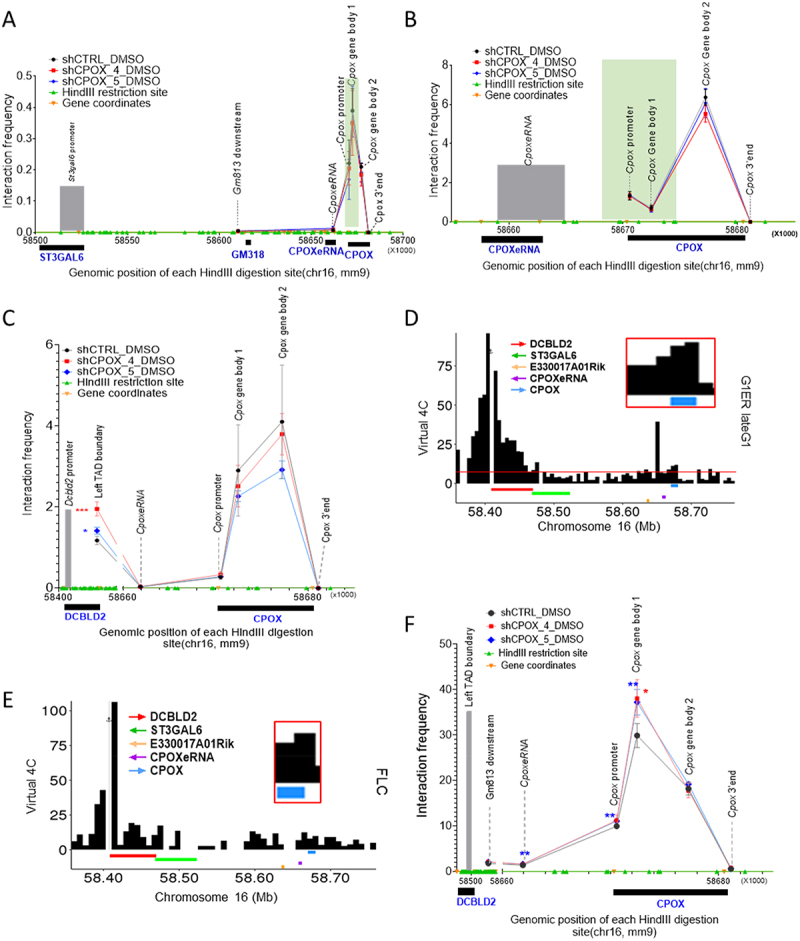
Chromatin interaction change after *Cpox* knockdown. A. 3C-qPCR result shows the interaction of *St3gal6* promoter with *CpoxeRNA* and *Cpox* locus in control and *Cpox* knockdown cells, *St3gal6* promoter as anchor. TAD boundary regions are highlighted in green. Three biological replicates. Data are mean ± s.D., unpaired two-tailed t-test. B. 3C-qPCR result shows the interaction of *CpoxeRNA* with *Cpox* locus in control and *Cpox* knockdown cells. *CpoxeRNA* locus as anchor. TAD boundary regions are highlighted in green. Three biological replicates. Data are mean ± s.D., unpaired two-tailed t-test. C. 3C-qPCR result shows the interaction of the *Dcbld2* promoter with the *Cpox* 3’ region, *Dcbld2* promoter as anchor. TAD boundary regions are highlighted in green. Four biological replicates for shCTRL, three biological replicates for shCPOX-4 and shCPOX-5. Data are mean ± s.D., unpaired two-tailed t-test. D. Virtual 4C plot from G1ER late G1 phase in situ HiC data shows the interaction between the *Dcbld2* promoter and *Cpox* 3’ region. *Dcbld2* promoter as anchor. E. Virtual 4C plot from foetal liver HiC data shows the interaction between *Dcbld2* promoter and *Cpox* 3’ region. *Dcbld2* promoter as anchor. F. 3C-qPCR result shows the interaction of TAD boundaries after *Cpox* knockdown. *Dcbld2* 3’end region (left TAD boundary) as anchor. TAD boundary regions are highlighted in green. Three biological replicates for shCPOX-4, four biological replicates for shCTRL and shCPOX-5. Data are mean ± s.D., unpaired two-tailed t-test. *:*p* ≤ 0.05; **:*p* ≤ 0.01, ***:*p* ≤ 0.001, ****: *p* ≤ 0.0001.

RNA was isolated with the RNeasy Plus Mini Kit (QIAGEN, catalogue no. 74134). 2 μg RNA was reverse transcribed into cDNA by SuperScript™ III First-Strand Synthesis System (Thermo Fisher, catalogue no.18080051) and then analysed by qPCR with iTaq Universal SYBR Green Supermix (Biorad, catalogue no. 1725124) using a QuantStudio 6 FLEX machine (Thermofisher). See Supplementary Data for primers. Actin was used as an internal control gene. Data were analysed with -∆∆Ct method for Figures 1(C), 6(F), 2^−∆Ct^ method for Figure 4(D) and supplementary Figure S3(a,b) and 2^−∆∆Ct^ method for the rest of the data [24,25].

### ChIP-qPCR

MEL cells differentiated 5 days in 2% DMSO were used for Chromatin immunoprecipitation as described [26]. Eluted DNA was analysed by qPCR with iTaq Universal SYBR Green Supermix (Biorad, catalogue no. 1725124) and QuantStudio 6 FLEX machine (Thermofisher). Antibodies used for immunoprecipitation were as follows: EZH2 (Cell Signaling, Catalogue #: 5246), H3K27me3 (Cell Signaling, Catalogue #: 9733), H3 (Abcam, Catalogue #: ab1791). For the p300 ChIP experiment, undifferentiated MEL cells were used, p300 antibody (Santa Cruz Biotechnology, Catalogue #: sc -48,343). See Supplementary Data for primers.

### 3C-qPCR

MEL cells differentiated for 5 days in 2% DMSO were used for 3C experiments performed as described [27,28]. DNA was analysed by qPCR with iTaq Universal SYBR Green Supermix (Biorad, catalogue no. 1725124) and QuantStudio 6 FLEX machine (Thermofisher). BAC controls were obtained from BACPAC company; alpha Aortic Actin-2(RP23-2N15), *Dcbld2-Cpox* locus (RP23-317L24, RP24-264K24, RP24–371018). See Supplementary Data for primers.

### DRIP-qPCR

Undifferentiated MEL cells were used for S9.6 (KeraFAST, ENH001) immunoprecipitation performed as described [14,29]. Eluted DNA was analysed by qPCR with iTaq Universal SYBR Green Supermix (Biorad, catalogue no. 1725124) and QuantStudio 6 FLEX machine (Thermo Fisher). See Supplementary Data for primers.

### Western blot

Protein was extracted with RIPA buffer in the presence of SuperScript™ Protease Inhibitor Cocktail (Thermofisher, catalogue no.78443) and quantified with MISSION® BCA Protein Assay Kit (Thermofisher, catalogue no. 23227). 20 μg protein was used for Western blotting. Antibodies used were as follows: EZH2 (Cell Signaling, 5246), H3K27me3 (Cell Signaling, 9733), ACT (Sigma, A3853), CPOX (Santa Cruz Biotechnology, sc -393,388), α-Tubulin (Santa Cruz Biotechnology, sc-5286).

### GSK343 treatment

Undifferentiated shCTRL and shCPOX-4 cells were seeded at 0.5 M cells/ml and treated with 5 μl 10 mm GSK343 (Sigma, catalogue no. SML076, final concentration 5 μM) or 5 μl DMSO (vehicle) for 24 hours.

### Data analysis

#### ChIP-seq data analysis

ChIP-seq data are all from ENCODE [30]. Bedtools (v.2.31.1) [31] slop was used to generate the extended H3K27ac region (±1kb around H3K27ac peak) file. Pybedtools [32] was used to find the overlapping region between p300 and extended H3K27ac region (±1kb around H3K27ac peak). Heatmap for H3K27ac, H3K27me3, and p300 and binding profiles of MYC, GATA2, and ELF1 around p300 only peaks were analysed by deeptools (v. 3.5.1) [33]. Pie chart of p300 only peak genome distribution was analysed by ChIPseeker (v. 1.18.0) [34]. Motif analysis was performed with HOMER (v. 4.11.1) [35].

#### Hi-C data analysis and generation of virtual 4C profiles

Contact matrix files (.hic) were obtained from the 4DN data portal [22,36] for Late G1 phase G1ER HiC (4DNFI6H926RO) and CH12.LX (4DNFI8KBXYNL). Figure 1(A) Foetal liver HiC raw Fastq files were obtained from [37], raw sequence data was mapped and processed using Juicer [38]. HiC matrix and virtual 4C were analysed by using GENOVA (dev version) [39]. Supplementary Figure S9(a) HiC matrices were obtained from 4DN data portal [22] and displayed by HiGlass [40]. Supplementary Figure S4(d), S5(a) and S9(a) insulation score and boundary tracks were obtained from 4DN data portal [22]. G1E-ER4 and CH12.LX RNA seq data were obtained from ENCODE. Supplementary Figure S3(c) processed HiC matrix was downloaded from GSE184974 [41], *Dcbld2-Cpox* TAD HiC map was analysed with GENOVA (dev version) [39].

#### Interaction categories, aggregate peak analysis and loop/TAD gene expression (Figure 5)

Loop and TAD files are obtained from G1ER cell cycle in situ HiC (GSE129997) [36]. For TAD, ±5kb region of each boundary was used for further analysis. Files from each cell cycle were merged by using HiCExplorer hicMergeLoops [42]. Interaction categories (Figure 5(E)) were identified with GenomicInteractions [43], promoter was defined as ±1kb around transcription start site while terminator was defined as ±5kb around the end of an annotated transcript. Lollipop plot was drawn with a home-made R script. Figure 5(F,G) aggregated peak analysis was done by using Juicer (v. 1.6) [38], with 25kb bins and window 6 normalized with VC_SQRT. G1ER in situ HiC late G1 phase.hic file (4DNFI6H926RO) was from 4DN database [22,36]. Figure 5(H) gene expression data was obtained from ENCODE (ENCFF199TJO.tsv) and was analysed with home-made R script and plotted with the ggpubr package.

#### TAD boundary interaction categories, gene expression, and oncoplot (Figure 6 and supplementary Figure S8 and S9)

TAD files were obtained from Rao S. et al., 2014 (GSE63525) [8], ±5kb region was used as TAD boundary for further analysis. Interaction categories (Figure 6(A) and Supplementary Figure S8(a)) were identified with GenomicInteractions [43], and Bar plot was drawn with ggpubr. Figure 6(B) and Supplementary Figure S8(b), gene expression data were obtained from ENCODE and were analysed with home-made R script and plotted with the ggpubr package. In Supplementary Figure S9(b), mutation at TSS was analysed with ICGC release 28 data by using script from Oh et al. [3], and Oncoplot was drawn with maftools [44].

### Quantification and statistical analysis

Statistical analysis for qPCR was carried out using both GraphPad Prism 9 Software and t.test function in Excel. Figures 5(H), 6(B), and Supplementary Figure S8(b) were using R, and detailed statistical methods were annotated in the figure legend. The types of the statistical tests and the exact value of ‘n’ are reported in the figure legends. qPCR results with biological replicates were combined from different plates as they were conducted in different batches, except for Supplementary Figure S2(b) and S3(a), where biological replicates were processed in parallel and qPCR was run on the same plate.

## Results

### Depletion of super enhancer derived pre-mRNA activates enhancer and neighbouring genes

The *Cpox* gene encodes the sixth enzyme in the haem synthetic pathway. HiC experiments using murine foetal liver erythroid cells show that *Cpox* is situated at the right boundary of a TAD that contains *Dcbld2*, *St3gal6*, *Gm813*, *Gm49701*, and *E330017A01Rik* [37] (Figure 1(A)). The *Dcbld2* gene, which has multiple isoforms, is located at the left TAD boundary. The promoter of the long isoform, *Dcbld2*-1, is situated outside the TAD boundary, while the promoter of the short isoform, *Dcbld2*-2, overlaps with the TAD boundary. According to annotation data from the dbSUPER super enhancer database [45] *Cpox* overlaps with a super enhancer (Figure 1(A)). Notably, the 5’ end of the super enhancer can be transcribed into an enhancer RNA. We named this RNA molecule *CpoxeRNA*. The promoter of *CpoxeRNA* is around 8kb away from the *Cpox* promoter, and, thus, it is not likely to be a PROMPT, which are closer [46]. Both *Cpox* mRNA and *CpoxeRNA* are upregulated during erythropoiesis (UMEL, undifferentiated MEL cells vs. iMEL, differentiated MEL cells) and *CpoxeRNA* shows an erythroid specific expression pattern (Figure 1(B) and Supplementary Figure S1(a)).

To dissect the function of the protein-coding gene, *Cpox*, within the super enhancer, we deleted its transcription start site (TSS) in MEL cells (Supplementary Figure S1(b-d)). After *Cpox* TSS deletion, *St3gal6* and *CpoxeRNA* were up-regulated and *Dcbld2–2* was down-regulated in both undifferentiated and differentiated cells, while *Dcbld2–1* was only decreased in differentiated cells (Figure 1(C), and Supplementary Figure S1(e) for primer locations). We next conducted experiments to knockdown (KD) *Cpox* using two different methods. First, we employed shRNAs targeting exon 5 (shCPOX-4) or exon 7 (shCPOX-5) of *Cpox*. In undifferentiated MEL cells (UMEL), *CpoxeRNA*, *St3gal6–1* and *Dcbld2*-2 were up-regulated by *Cpox* KD, while *Cldnd1*, which is located outside of this TAD, was not affected (Figure 1(C)). In differentiated MEL cells (iMEL) with *Cpox* KD, *St3gal6* and *CpoxeRNA* were upregulated, as well as both isoforms of *Dcbld2*. This result was confirmed by a different knockdown method using anti-sense oligonucleotides (ASO) to target intron 1 or intron 5 of *Cpox* (Figure 1(C)).

To determine whether the effects observed after *Cpox* knockdown (KD) are due to the loss of pre-mRNA, mature mRNA, or protein, we designed an shRNA targeting the exon–exon junction of *Cpox* mature mRNA. We found that the shRNA targeting the junction between exon 2 and exon 3 (shCPOX-EXJ2/3) effectively reduced both RNA and protein levels (Supplementary Figure S2(a)). To control for any isoform-specific effects, we also designed an shRNA targeting exon 2, which partially overlaps with shCPOX-EXJ2/3. As shown in Supplementary Figure S2(b), the shRNA targeting exon 2 recapitulated the effects of shRNA4 and shRNA5, whereas shCPOX-EXJ2/3 did not. This result suggests that the observed effects after *Cpox* KD are due to the loss of pre-mRNA. To further confirm this, we expressed the *Cpox* ORF in both shCTRL and *Cpox* KD cells and found that expressing the *Cpox* ORF could not rescue the KD effect (Supplementary Figure S3(a)). These findings confirm that the loss of pre-mRNA is responsible for the upregulation of enhancer RNA and neighbouring genes.

The difference of *Dcbld2* expression between *Cpox* TSS deleted cells and *Cpox* KD cells suggests that the *Cpox* TSS, which overlaps with the super enhancer, functions as the enhancer for *Dcbld2*. The TAD corner interaction modestly increased after differentiation, coinciding with the upregulation of the long isoform of *Dcbld2* (*Dcbld2–1*), suggesting that the intact TAD structure is essential for *Dcbld2* expression (Supplementary Figure S3(b,c)). Notably, *Cpox* KD did not affect erythropoiesis as judged by the production of haemoglobin and erythroid cell surface markers upon differentiation (Supplementary Figure S4(a, b)). This observation is consistent with a previous study [47] reporting that *Cpox* absence can be bypassed in erythropoiesis. Our qRT-PCR analysis of nascent RNA in undifferentiated MEL cell confirmed that the upregulation of *Dcbld2*, *St3gal6*, and *CpoxeRNA* following *Cpox* knockdown was due to increased transcription (Supplementary Figure S4(c)). Overall, these results suggest that the pre-mRNA transcribed from *Cpox* within a super enhancer has a non-coding function in regulating the expression of neighbouring genes. Furthermore, our findings provide evidence that the pre-mRNA transcribed from the super enhancer can act as a repressor, while its genomic locus functions as an enhancer.

### Cpox knockdown affects intra-TAD interactions

To explore the effect of *Cpox* knockdown on intra-TAD interactions, we first focused on the only protein coding gene located inside of this TAD— *St3gal6*. We performed a 3C experiment using the *St3gal6* promoter as anchor, since 3C has the highest resolution and is without bias present in capture-based techniques. We found the *St3gal6* promoter interacts most strongly with the *Cpox* gene body 1 region, which overlaps with the core region of the TAD boundary. The interactions between the *St3gal6* promoter and *Cpox* promoter/gene body regions are all modestly reduced in the *Cpox* knockdown cells (Figure 2(A)).

Next, we checked the intra-super enhancer interactions. *CpoxeRNA* shows the highest interaction with the *Cpox* gene body 2 region, and this interaction decreased after *Cpox* KD (Figure 2(B)). These results demonstrate that knocking down *Cpox* pre-mRNA can promote release of *St3gal6* and *CpoxeRNA* from *Cpox* gene body interactions, which correlates with transcription up-regulation, supporting that the gene body region of *Cpox* functions as a repressor.

### Cpox knockdown alters interaction between TAD boundary genes

The *Dcbld2* gene is located at the left TAD boundary, with the 3’ region overlapping the TAD boundary, which contains tandem CTCF binding sites (Supplementary Figure S4(d)). The *Cpox* gene is located at the right TAD boundary, with the 5’ region overlapping the boundary but without a CTCF binding site (Supplementary Figure S4(d)). Since a promoter can act as the anchor of a TAD [10,48], it is possible that the right TAD boundary is anchored by the promoter of *Cpox*. To investigate *Dcbld2*/left TAD boundary long-range interactions, we first set the anchor for 3C at the *Dcbld2* promoter and found that the *Dcbld2* promoter has a higher interaction frequency with the gene body region of *Cpox* than with the *Cpox* promoter and termination site, especially with gene body region 2 (Figure 2(C)). Gene body region 2 contains the 3C fragment that extends from the end of exon 4 and includes most of the last (7^th^) exon. This pattern is confirmed by virtual 4C analysis of cell cycle in situ HiC data in G1ER undifferentiated erythroid cells and in situ HiC in foetal liver erythroid progenitor cells [22,36,37] (Figure 2(D,E)).

After knocking down *Cpox*, the interaction between the *Dcbld2* promoter and the *Cpox* gene body region 1 and 2 decreased, whereas the interaction of *Dcbld2* promoter with the left TAD boundary significantly increased (Figure 2(C)). This left TAD boundary 3C fragment overlaps with the 3’ end of the *Dcbld2* gene. These results suggest that the *Dcbld2* promoter was sequestered by the gene body region of *Cpox*, and after *Cpox* pre-mRNA loss, the *Dcbld2* promoter is released and has increased interaction with its own transcription termination site (TTS). Furthermore, the promoter interaction profiles of *Dcbld2*, *St3gal6* and *CpoxeRNA* suggest that they are directly regulated by *Cpox*. Therefore, the mechanism underlying their activation after *Cpox* KD differs from the Enhancer Release and Retargeting (ERR) model [3] in which the neighbouring genes are not regulated directly by the protein coding gene.

To investigate TAD organization in more detail, we used the left TAD boundary as an anchor for a 3C experiment. Our analysis revealed that the left TAD boundary region had the highest interaction frequency with the *Cpox* gene body region 1, which overlaps with the right TAD boundary, and this interaction was significantly increased after *Cpox* knockdown (Figure 2(F)). This suggests that loss of *Cpox* pre-mRNA can reinforce TAD corner interaction. Notably, the *Cpox* gene body region 1, located at the right TAD boundary, overlaps with H3K27ac marks. The increased interaction with the left TAD boundary (*Dcbld2* 3’ region) brings the *Dcbld2* 3’ region to a conducive environment for transcription. Simultaneously, the increased interaction of the *Dcbld2* 3’ region with its own promoter positions the *Dcbld2*-1 promoter closer to this positive environment, potentially facilitating the transcription of *Dcbld2* long isoform (*Dcbld2–1*). Taken together, these results support that the TAD anchor genes *Dcbld2* and *Cpox* are interconnected head to tail. TAD anchors are typically organized with convergent CTCF sites. However, the conformation of TAD anchors that are not occupied by CTCF is not well understood. Here, we present an example of a TAD anchored by two genes that lie in the same direction along the linear genome and are organized in a head-to-tail inter-gene connection to anchor the TAD.

### Increased transcriptional R loop formation strengthens TAD boundary insulation

R loops have been reported to play a role in mediating TAD boundary interactions [14]. To investigate whether R loop formation mediates the increased TAD boundary interaction observed after *Cpox* knockdown, we utilized an R loop-specific antibody, S9.6, to perform DNA-RNA immunoprecipitation (DRIP) experiment with the addition of RNaseH *in vitro* to digest RNA-DNA hybrids during the pull-down procedure as control. We found that R loop formation was increased at both TAD boundaries after *Cpox* knockdown (Supplementary Figure S5(a)). The increase in R loop formation in *Dcbld2* is correlated with up-regulation of its transcription. This suggests that transcriptional activation may mediate the formation of transcriptional R loops, ultimately leading to the strengthening of the TAD corner interaction.

To further confirm the role of increased transcription in strengthening the TAD boundary interaction at the *Dcbld2* locus, we examined 4DN HiC data from other cell types and observed that CH12.LX cells, which also have this TAD, exhibit lower corner loop interaction compared to G1ER erythroid cells [22] (Supplementary Figure S5(b)). RNA-seq data revealed lower *Dcbld2* and *Cpox* expression levels in CH12.LX cells compared to G1ER cells (Supplementary Figure S5(c)). These results support the notion that increased TAD boundary transcription correlates with increased TAD corner interaction.

### Perturbed p300 binding at Cpox intron 5 contributes to boundary gene Dcbld2 and intra-tad enhancer activation

**Figure 3.**
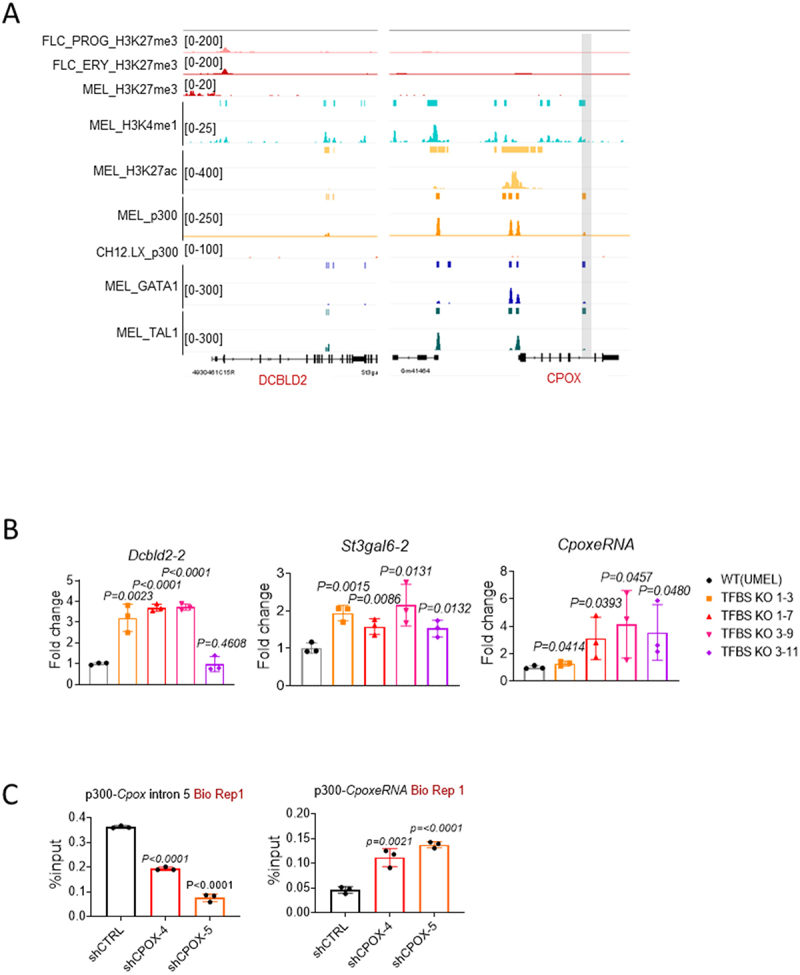
p300 binding at *Cpox* intron 5 contributes to the regulation of intra-tad enhancer and TAD boundary gene *Dcbld2*. A. UCSC genome browser tracks shows p300, GATA1, TAL1, H3K27ac, H3K27me3, and H3K4me1 pattern at the *Dcbld2* promoter and *Cpox* intron 5. H3K27me3 ChIP-seq data for E14.5 foetal liver erythroid progenitor cells (FLC_PROG) and erythroblast cells (FLC_ERY) are from Alvarez-Dominguez et al. [21]. All the other ChIP-seq data are from ENCODE. p300 only peak was highlighted in grey. CH12.LX p300 track was used to show *Cpox* intron 5 p300 peak is cell type specific. B. qRT-pcr result shows the expression of *Dcbld2–2*, *St3gal6–2* and *CpoxeRNA* after *Cpox* intron 5 TFBS deletion in UMEL cells. Two pairs of sgRNA are shown (pair1, pair3), and each has two different clones (clone 3 and 7 for sgRNA pair1, and clone 9 and 11 for sgRNA pair3). Three biological replicates. Data are mean ± s.D., unpaired one-tailed t-test. C. ChIP-qPCR result shows p300 binding inside TAD after *Cpox* knockdown by shRNA in UMEL cells. Three independent experiments, one representative result shown here. Data are mean ± s.D., unpaired one-tailed t-test.

The 3C fragment located in the 3’ region of *Cpox* interacts with both the *Dcbld2* promoter and *CpoxeRNA*. It spans from exon 4 of *Cpox* to include most of its last exon. Intron 5 of *Cpox* contains binding sites for known transcription factors (TFBS) that can regulate transcription termination [49], including GATA1, TAL1 and p300 (Figure 3(A)). We reasoned that intron 5 TFBS could play an important role in regulating *Dcbld2* and *CpoxeRNA* expression. To test this hypothesis, we used CRISPR-Cas9 to delete intron 5 TFBS (Supplementary Figure S6(a-c)). After intron 5 TFBS deletion, the mRNA and protein levels of *Cpox* exhibited different patterns in different single-cell clones, as shown in Supplementary Figure S7(a,b). Clonal variability may be due to varying stability of the mRNA after the different length of TFBS deletions or due to heterogeneity of the wild type MEL cell population [50]. However, *Dcbld2*, *St3gal6* and *CpoxeRNA* were consistently up-regulated (Figure 3(B)), indicating that intron 5 TFBS has an inhibitory role. This result further confirms that activation of *Dcbld2*, *St3gal6* and *CpoxeRNA* after *Cpox* knockdown is due to the loss of looping with the intron 5 locus and not due to an altered level of CPOX protein.

Next, we investigated which protein is responsible for the repression of intra-TAD neighbouring genes and enhancer. By examining the ENCODE ChIP-seq data in MEL cell, we found a p300 peak at the intron 5 TFBS that overlaps with H3K4me1, but not with H3K27ac (p300 only peak, Figure 3(A)). Regulatory elements with H3K4me1 but without H3K27ac were described as poised enhancers. Some poised enhancers also contain H3K27me3 [51–54], while other regulatory elements with H3K4me1 and H3K27me3 were considered silencers [55]. According to our KO experiment result, the intron 5 element behaves like a repressor but without overlap with H3K27me3.

p300 catalyses and deposits H3K27ac, which is a hallmark active epigenetic mark [56,57]. Recent research has suggested that p300 can also occupy the H3K27me3 region at poised enhancers [51,52,58]. Additionally, it has been reported that EZH2, a key component of the polycomb repressive complex 2 (PRC2), interacts with p300 [59]. Notably, p300/CBP is crucial for PRC2 silencing in regions that are co-occupied by CBP/PRC2, independent of its enzymatic activity [60]. The role of p300 occupancy in the absence of H3K27ac modifications is not fully understood. ENCODE histone ChIP-seq data revealed that the *Dcbld2* promoter is enriched in H3K27me3 in MEL cells (Figure 3(A)). Given that the *Dcbld2* promoter and *Cpox* intron 5 TFBS interact, we postulated that this association may be facilitated by PRC2-p300 and that p300 bound at *Cpox* intron 5 should have a repressive role due to its association with PRC2. If this assumption is valid, we would expect that p300 binding at the *Cpox* intron 5 TFBS would diminish following *Cpox* pre-mRNA depletion. Our findings confirm this hypothesis, as evidenced by the observed reduction in p300 binding at the *Cpox* intron 5 TFBS after *Cpox* knockdown (Figure 3(C) and Supplementary Figure S7(c)).

The observed decrease in p300 binding at *Cpox* intron 5 following *Cpox* knockdown implies the release of p300 from the *Cpox* intron 5 locus. This event may subsequently lead to an increase in the concentration of activators in the insulated TAD environment (Figure 3(C)), thereby activating enhancers and protein coding genes located in the vicinity. p300 release may also explain the activation of intra-TAD genes (*St3gal6*) and enhancer after *Cpox* TSS deletion.

### H3K27me3 accumulation at the Cpox locus mediates the activation of neighbouring genes

**Figure 4.**
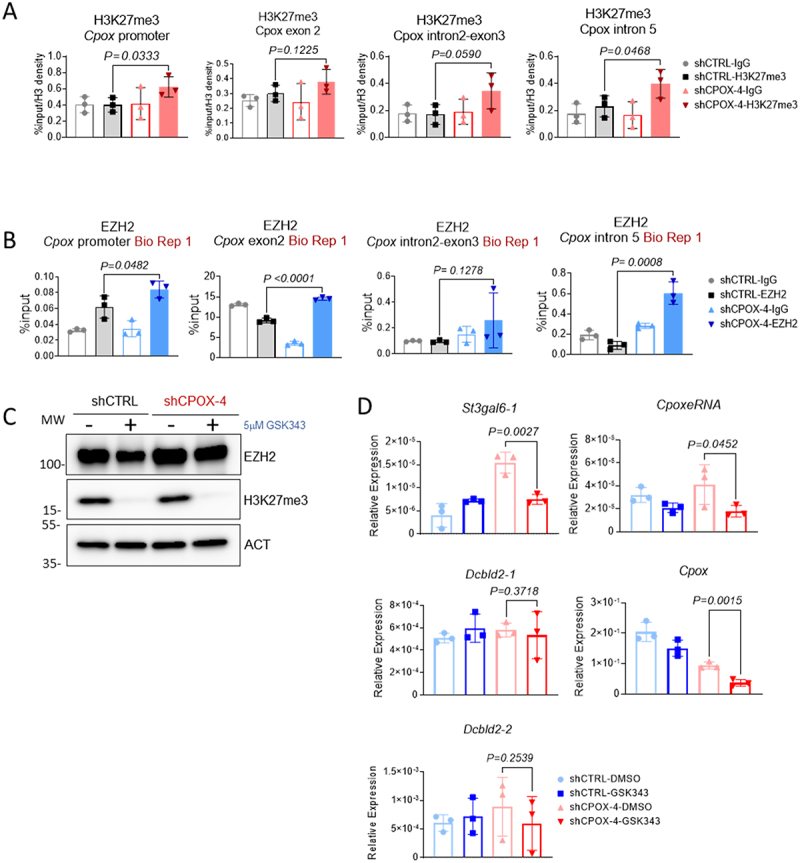
H3K27me3 and PRC2 translocation after loss of *Cpox* pre-mRNA. A. ChIP-qPCR result shows H3K27me3 accumulation at the *Cpox* locus after *Cpox* knockdown in iMEL cells. Three biological replicates. Data are mean ± s.D., unpaired one-tailed t-test. B. ChIP-qPCR result shows EZH2 accumulation at the *Cpox* locus after *Cpox* knockdown in iMEL cells. Three independent experiments, one representative result shown here. Data are mean ± s.d., unpaired one-tailed t-test. C. Western blot result shows depletion of H3K27me3 after 24 h GSK343 treatment. D. qRT-pcr result shows gene expression change after 24 h GSK343 treatment in UMEL. Vehicle is treated with DMSO only. Statistical test was performed with dmso-shCPOX-4 and GSK343-shCPOX-4 samples. Three biological replicates. Data are mean ± s.D.; unpaired one-tailed t-test.

Next, we investigated the mechanism underlying the perturbed looping and TF/co-activator binding at the *Cpox* locus after *Cpox* knockdown. It has been reported that RNA competes with chromatin to bind PRC2, and TSS loss results in the accumulation of H3K27me3 in chromatin [4,5]. To test whether H3K27me3 accumulation is involved in the activation of neighbouring genes and *CpoxeRNA*, we used ChIP-qPCR to detect changes in the H3K27me3 levels at the *Cpox* locus after *Cpox* knockdown (see supplementary Figure S6(d) for primer locations). As shown in Figure 4(A), H3K27me3 accumulated at the *Cpox* genomic locus after *Cpox* KD, with especially significant increases at the *Cpox* promoter and intron 5 regions. EZH2 also tended to be higher across *Cpox* after pre-mRNA loss (Figure 4(B) and Supplementary Figure S7(d)). These results suggest that after *Cpox* pre-mRNA loss, PRC2 accumulates at the *Cpox* genomic locus and this correlates with the acquisition of the repressive H3K27me3 mark across *Cpox*.

To further confirm that the activation of neighbouring genes is due to PRC2 mediated repression of the *Cpox* genomic locus, we treated the control and *Cpox* knockdown cells with PRC2 inhibitor GSK343, and western blot analysis confirmed the drug’s effect as H3K27me3 levels were nearly completely depleted (Figure 4(C)). After GSK343 treatment, the levels of *CpoxeRNA* and neighbouring gene *St3gal6* dropped to control levels and *Dcbld2–2* exhibited a decreased trend, consistent with the idea that their up-regulation after *Cpox* pre-mRNA knockdown is due to PRC2 repression of the *Cpox* genomic locus (Figure 4(D)).

### Genome-wide discovery of p300-H3K27me3 loops unveils their repressive role in regulating gene expression

**Figure 5.**
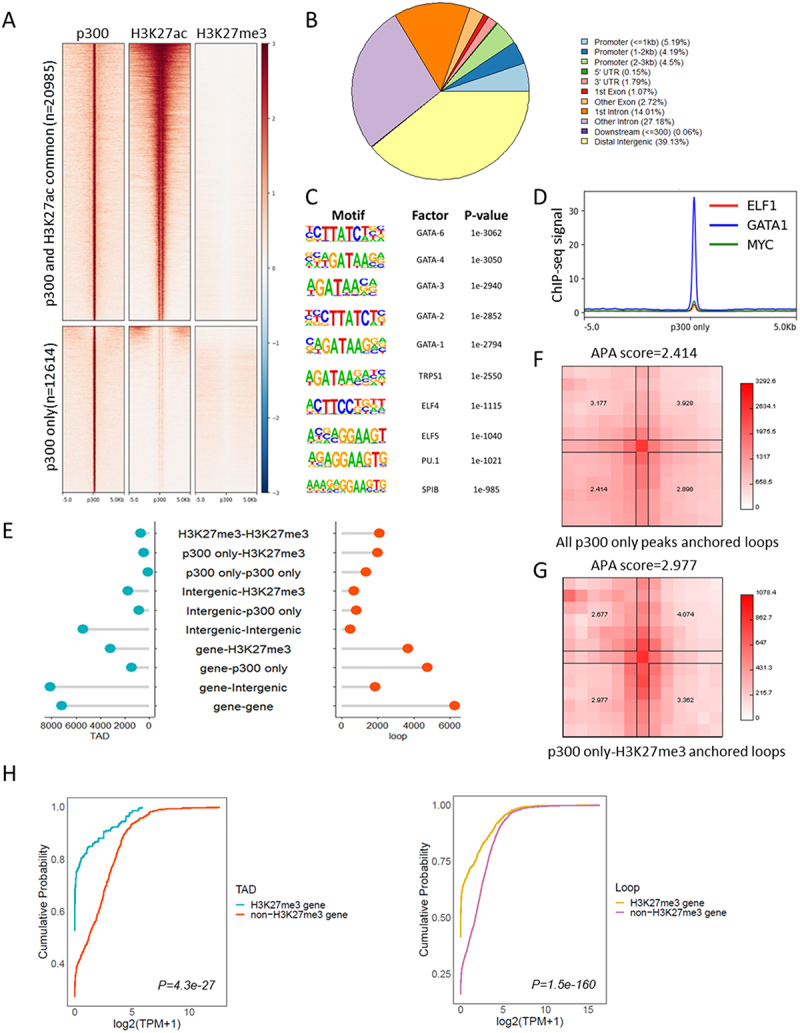
Genome wide discovery of p300-H3K27me3 repressive loop. A. Heatmap shows the distribution of p300, H3K27ac and H3K27me3 signals around ± 5kb region of p300 only peak and p300/H3K27ac common peaks. B. Pie chart shows the distributions of annotated features around p300 only peaks in MEL cell. C. Homer motif analysis identifies significant enrichment of transcription factors binding sites at p300 only peaks. D. Binding profiles of transcription factors at p300 only peak in MEL cells. E. Distribution of different types of interaction observed at TAD boundary (blue) and chromatin loops (red). X axis represents number of different types of interactions. ‘Gene’ represents genes that do not overlap with H3K27me3 and p300 only peak. ‘Intergenic’ represents intergenic regions that do not overlap with H3K27me3 and p300 only peak. F and G. Aggregated peak analysis (APA) of chromatin loops anchored by p300 only peaks (F) and p300 only peak-H3K27me3 peaks (G) in G1ER late G1 cell. HiC matrix was normalized with VC_SQRT, window = 6. H. ECDF plot shows the comparison of the expression level of H3K27me3 non-overlapping and overlapping genes looped to p300 only peaks at TAD boundary or chromatin loops. MEL cell RNA-seq processed expression quantification data was obtained from ENCODE. Wilcoxon test was used to compare the log_2_(TPM +1) value of H3K27me3 genes and non-H3K27me3 genes, *p* values ≤ 0.05 indicating a significant difference.

To determine the prevalence of p300-PRC2/H3K27me3 loops across the genome, we analysed ENCODE MEL cell ChIP-seq profiles for the distribution of H3K27me3 and extended H3K27ac (±1kb region) peaks around p300 binding sites. We found that a subset of p300 binding sites did not exhibit occupancy by H3K27ac (Figure 5(A)). Notably, both the H3K27ac overlapping peaks and the H3K27ac independent peaks were devoid of H3K27me3 binding. To further explore this phenomenon, we focused on p300 peaks that did not overlap with the extended H3K27ac peaks (±1kb region). These H3K27ac-independent p300 peaks were predominantly localized in intergenic and intronic regions, with a notable preference for other introns besides the first intron (Figure 5(B)). Given that p300 has diverse targets beyond histones, we performed motif analysis using HOMER to identify the transcription factors co-localized at p300-only peaks. We observed a significant enrichment of cell type-specific transcription factors, including primarily GATA factors, but also ELF factors and PU.1 (Figure 5(C)). To validate these findings, we examined the binding profiles of several transcription factors, confirming the enrichment of cell-type-specific transcription factors at the p300-only peaks (Figure 5(D)).

To identify the loops anchored by both p300 and PRC2/H3K27me3 on a genome-wide scale, we analysed published G1ER cell cycle Hi-C data [22,36]. We found that 1.52% of TAD boundary interactions and 8.33% of loops were anchored by p300-only and H3K27me3 peaks (Figure 5(E)). The formation of loops anchored by all the p300-only peaks and p300-only-H3K27me3 peaks was confirmed through aggregated peak analysis (APA) (Figure 5(F,G)).

To investigate whether p300-only-H3K27me3 anchored loops have a repressive role, we compared the expression levels of genes that form loops with p300-only peaks. Notably, genes overlapping with H3K27me3 exhibited significantly lower expression levels compared to those without H3K27me3 peaks (Figure 5(H)). This finding confirms that p300-H3K27me3/PRC2 loops are associated with gene repression. Thus, we suggest that p300-H3K27me3 loops could serve as a mechanism for protein-coding genes to repress their interacting protein-coding counterparts, providing insight into the regulatory mechanisms underlying gene expression.

### Knockdown of non-oncogenes in a head-to-tail TAD boundary gene pair activates its partner oncogene

**Figure 6.**
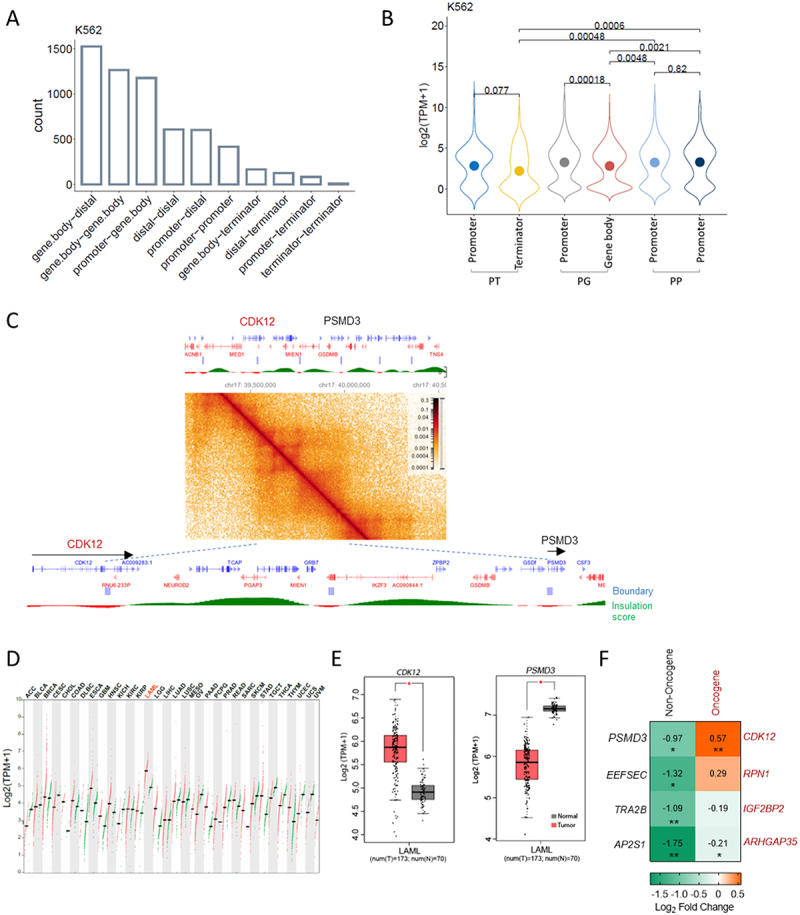
Head-to-tail TAD boundary gene conformation is a potential mechanism to restrain the activation of certain oncogenes. A. Distribution of different types of interactions observed at TAD boundary in K562 cells. Y axis shows the number of the TAD. B. Expression level at each TAD boundary for promoter-terminator (PT), promoter-genebody (PG) and promoter-promoter (PP) boundary pairs in K562 cells. Dots inside of the violin plots show the mean value. Kruskal-Wallis test, *p* values ≤ 0.05 indicating a significant difference. C. *CDK12-PSMD3* TAD in K562 cell. Data from 4DN portal [8,22]. D. Dot plot shows the expression level of *CDK12* across various tumour samples (red) and paired normal tissues (green). Data from GEPIA [23]. AML is highlighted in red. E. Expression level of *CDK12* and *PSMD3* in AML samples and paired normal tissues. Data from GEPIA [23] with Log_2_FC cut-off as 0.5, *p* value cut-off as 0.05. F. Expression level of TAD anchor oncogenes after knocking down its paired TAD anchor non-oncogene genes in 293T cell. Two biological replicates, unpaired one-tailed t-test. *: p ≤ 0.05; **: p ≤ 0.01, ***: p ≤ 0.001, ****: p ≤ 0.0001.

We explored whether regulation by head-to-tail conformation of boundary genes is pervasive and whether it could exist beyond super enhancers. Since the TAD is a conserved structure, we extended our analysis to different cell types and species. We examined the interaction patterns of TAD boundary genes in human K562 cells and GM12878 cells, and mouse CH12.LX cells and G1ER cells. We found that the most enriched interaction patterns among TAD boundary gene pairs are gene body−gene body interaction and promoter-gene body interaction (PG) (Figure 6(A) and Supplementary Figure S8(a)). When comparing the expression of promoter-gene body (PG), promoter-terminator (PT) and promoter–promoter (PP) gene pairs, genes with their gene body or terminators overlapping with a TAD boundary were expressed at a lower level than genes having their promoters overlapping with TAD boundary (Figure 6(B) and Supplementary Figure S8(b)). This is exemplified by the *Dcbld2-Cpox* pair: the *Dcbld2* gene body overlaps with the TAD boundary and is expressed at a lower level than *Cpox*, whose promoter overlaps with the partner boundary. This indicates that head-to-tail inter-gene interaction functions to restrict gene expression.

Next, we explored whether the head-to-tail inter-gene interaction plays any role to repress oncogene activation. One example oncogene/non-oncogene pair is shown in Figure 6(C), *CDK12-PSMD3*, which has the same conformation as the *Dcbld2-Cpox* pair (i.e. in the same direction on the linear genome while the upstream gene 3’ region and the downstream gene promoter overlap with a TAD boundary) with the gene body of *CDK12* and the promoter of *PSMD3* overlapped with a TAD boundary. *CDK12* is an oncogene, which is up-regulated and has the highest expression level in Acute Myeloid Leukemia (AML) (Figure 6(D)), while its boundary partner non-oncogene *PSMD3* shows down-regulation in AML patients (Figure 6(E)). To further validate whether there is a causal relationship between decreased expression of *PSMD3* and increased expression of *CDK12*, we performed a *PSMD3* shRNA knockdown experiment in 293T cells (Figure 6(F)). Indeed, *CDK12* was activated after *PSMD3* KD. Given that *PSMD3* and *CDK12* share a similar conformation as *Dcbld2* and *Cpox*, this suggests that the promoter of *CDK12* is released from *PSMD3* after *PSMD3* knockdown and the activators associated with *PSMD3* are liberated into the TAD, providing additional support for the up regulation of *CDK12*.

To further validate this concept, we selected three additional pairs of non-oncogene/oncogene, each with the non-oncogene’s promoter and the gene body/terminator of the oncogene overlapping a TAD boundary but exhibiting distinct conformations compared to *Dcbld2-Cpox*. We performed knockdown experiments with shRNA in 293T cells. As shown in Figure 6(F), after knocking down the non-oncogenes, only one showed up-regulation of its oncogene partner but without statistical significance. These non-oncogene/oncogene pairs are not in the same conformation as *Dcbld2*/*Cpox* pair (i.e. in the same direction on the linear genome while the upstream gene 3’ region and downstream gene promoter overlap with a TAD boundary) (Supplementary Figure S9(a)). This suggests that head-to-tail boundary gene conformation is required for a downstream non-oncogene to suppress an upstream oncogene.

We identified 358 TADs in K562 cell that are in the conformation like *Dcbld2*/*Cpox*. 2.3% of the oncogenes in these pairs have their gene body/terminator overlapped with the TAD boundaries and are potentially repressed by their downstream partner non-oncogenes, which have promoter overlap with the TAD boundary. We checked the mutation distribution at promoters of TAD boundary non-oncogenes by analysing data from the International Cancer Genome Consortium (ICGC). Oncoplots showed that in 1% of about 6000 cancer samples, the non-oncogenes have mutations at their promoters (Supplementary Figure S9(b)). These results suggest that head-to-tail inter-gene interaction could be a mechanism for cells to restrain the expression of a subset of oncogenes.

## Discussion

Our findings demonstrate a novel non-coding function of pre-mRNA, revealing its regulatory role in neighbouring gene expression and chromatin structure. As proposed in the model in Supplementary Figure S9(c), *Cpox* pre-mRNA loss results in an accumulation of H3K27me3 at the *Cpox* genomic locus, thereby altering its chromatin looping pattern with neighbouring genes. On the one hand, both the TSS and TTS regions of the TAD boundary gene *Dcbld2* interact with *Cpox* connected in an inter-gene TSS-TTS loop conformation. Following *Cpox* pre-mRNA depletion, the *Dcbld2* TSS-*Cpox* TTS loop decreased while the *Dcbld2* TSS- *Dcbld2*-TTS loop increased. This release of the *Dcbld2* TSS from the *Cpox* TTS allows it to connect with its own TTS, thus facilitating its transcription. Furthermore, the increased transcription leads to the formation of transcriptional R loops, which, in turn, strengthen the TAD corner loop. On the other hand, p300 is released from *Cpox* intron 5 due to the accumulation of H3K27me3, resulting in an increased availability of p300 in the TAD insulated environment (Figure 3(C)). Elevated p300 availability correlates with the activation of enhancer and protein coding genes within the TAD.

In summary, our results reveal that pre-mRNA can play a crucial role in gene regulation, particularly in chromatin looping patterns and enhancer activation. These findings shed light on a previously unknown mechanism of gene regulation and highlight the importance of understanding the complex interplay between pre-mRNA and chromatin structure.

Among the individual enhancers making up a super enhancer, there is a hierarchy in that these elements may have variable enhancer activity [61–63]. For example, super enhancer components can be divided into hub enhancers and non-hub enhancers, with hub enhancers associated with higher levels of cohesin and CTCF binding [61]. For protein coding gene-containing super enhancers, the contribution to enhancer activity of a protein coding gene versus a non-coding enhancer element remains unexplored. Our results demonstrating that in the super enhancer containing the protein coding gene *Cpox* and non-coding *CpoxeRNA*, the protein coding gene seems to have a dominant role over the non-coding gene. Specifically, the protein coding gene *Cpox* represses the non-coding enhancer.

Many studies have focused on how enhancers could regulate gene expression, while less attention has been paid to how the enhancer could be regulated. Our results provide an example showing that an enhancer can be regulated and harnessed by a protein coding gene and the pre-mRNA it encodes. The ERR model shows that the activation of an enhancer after promoter repression is due to loss of interaction with the promoter [3]. However, in our case, we observed *CpoxeRNA* mainly interacts with the gene body region of *Cpox* and the interaction between *CpoxeRNA* and the *Cpox* promoter is unchanged following *Cpox* RNA loss. Within the confines of the TAD we studied, *Cpox* pre-mRNA loss could result in locally increased free PolII or another transcription activator, which will then have an increased chance to bind with the *CpoxeRNA* promoter and drive its expression. Our results also indicate a decrease in p300 at the *Cpox* intron 5 locus, suggesting that p300 could be a potential activator of *CpoxeRNA* after *Cpox* pre-mRNA knockdown. This may indicate p300 at *Cpox* intron 5 has a repressive role to harness *CpoxeRNA* expression and supports that the effect we see upon *Cpox* pre-mRNA deletion is due to relief of repression by the intron 5 TFBS region.

We uncovered a new type of loop anchored by H3K27ac-independent p300 and PRC2/H3K27me3. Although this type of loop comprises less than 10% of all chromatin loops genome-wide, it confers cell-type specific chromatin looping and regulation of gene expression, since p300 only peaks and PRC2/H3K27me3 are variable in different cells. Our results suggest that this loop could be mediated by RNA, as depletion of *Cpox* pre-mRNA reduces the frequency of this chromatin interaction.

The spatial proximity of p300 and PRC2 may allow p300 to stabilize PRC2 at the *Dcbld2* promoter. We propose this hypothesis based on the observed deletion of the intronic p300-only site, we think that the mere knockdown or knockout of the p300 gene might not faithfully elucidate the role of such p300-only peaks. In the illustrative case presented here, both *Dcbld2* and *CpoxeRNA* exhibit transcription in wild-type (WT) cells, with their expression further increasing in cells with a deleted p300-only binding site. *CpoxeRNA* and *Dcbld2* overlaps with p300/H3K27ac common peak. This suggests that the binding of p300/H3K27ac at their loci plays a supportive role in their expression. Conversely, the p300-only peak at *Cpox* intron 5, identified as a potential silencer element, loops to target genes to repress and curtail their expression. A global depletion of p300 would result in the loss of p300/H3K27ac common peaks in these target genes, potentially impairing their expression. Consequently, the true impact of the p300-only peak at *Cpox* intron 5 might be obscured. A global depletion of p300 would be more likely to reveal the function of p300-only peaks in those target genes whose expression is independent of p300.

Our results support that the mechanism for repression of *Dcbld2* by *Cpox* is sequestration of the *Dcbld2* promoter by 3’ region of *Cpox*, and the sequestration is mediated by a novel type of repressive chromatin loop established by p300 and PRC2/H3K27me3. Promoter sequestration could be mediated by different looping proteins, for instance, we observed that gene pairs like *PSMD3-CDK12* exist in TSS−TTS interaction conformation but do not possess a p300 binding site (Figure 6). This also shows that Head-to-Tail inter-gene interaction could be clinically important. As knockdown of TAD anchor non-oncogenes *PSMD3* led to activation of its partner boundary oncogene *CDK12*, RNA mutation of the non-oncogene could be a potential mechanism that underlies cancer driver gene activation. Chromatin 3D conformation alteration in cancers may create new TAD boundaries that overlap with oncogenes or tumour suppressor genes to activate or suppress their expression. We also found around 2% of tumour suppressor genes are located in TAD boundaries that connected to downstream partner boundary genes in head-to-tail conformations. We speculate that these tumour suppressor genes could be re-activated by knockdown of its partner TAD boundary gene in cancer samples, which could be a RNAi-based novel therapeutic approach for cancer.

## Limitations of the study

Our study highlights a specific role for *Cpox* pre-mRNA in regulating chromatin architecture and neighbouring gene and enhancer expression within the *Dcbld2-Cpox* TAD. The evidence presented here suggests that pre-mRNAs transcribed from super enhancer regions may play functional roles in chromatin organization and gene regulation. However, our findings are limited to a single super enhancer locus and cell type. Further research is required to validate these mechanisms across different genomic loci and biological systems. Genome-wide approaches and additional functional assays will be particularly valuable in determining whether these observations can be generalized or are specific to certain super enhancer contexts.

Some of the qPCR results in our study exhibit relatively high p-values because biological replicates from different experimental batches were used. Batch effects, such as processing samples at different times and running qPCR on separate plates, can inflate variability and affect statistical significance. Using more biological replicates or processing samples in parallel on the same qPCR plate could help reduce batch effects and improve consistency (Supplementary Figure S2(b)). However, we acknowledge that genome-wide approaches, such as nascent RNA sequencing (5EU-seq or PRO-seq) or ChIP-seq, would provide a more comprehensive and statistically robust assessment of RNA and chromatin changes.

While we provide the *PSMD3-CDK12* example that highlights the potential clinical relevance of head-to-tail TAD boundary gene arrangements, further identification and functional validation of additional examples of head-to-tail TAD boundary gene arrangements can help to better understand their regulatory and clinical significance.

## Supplementary Material

Supp.docx

## Data Availability

Figure 1(A) Mouse foetal liver HiC data Fastq files were downloaded from EBI ArrayExpress (https://www.ebi.ac.uk/arrayexpress/) accession number E-MTAB-2414. Supplementary Figure S3(c) mouse foetal liver progenitor and differentiated HiC process data were downloaded from GSE184974. 4DN .hic file used in this study is downloaded from 4DN data portal: G1ER in situ cell cycle HiC late G1 phase (4DNFI6H926RO), CH12.LX (4DNFI8KBXYNL), and K562 in situ HiC (4DNFI18UHVRO). Cell cycle G1ER in situ HiC boundary files and insulation score files are downloaded from 4DN data portal [22,36]. TAD files and loop files for G1ER cell were downloaded from GSE129997. TAD files for K562 cell, GM12878 cells and CH12.LX cells were downloaded from GSE63525. RNA-seq gene quantification files were downloaded from ENCODE: MEL cell (ENCFF199TJO.tsv), K562 cell (ENCFF421TJX.tsv), GM12878 cell (ENCFF345SHY.tsv), CH12.LX cell (ENCFF164NIW.tsv), G1ER cell (ENCFF608FAD.tsv). ChIP-seq data for MEL cell H3K27ac (ENCFF972YIT.bed.gz and ENCFF847MJA.bigWig), p300 (ENCFF185UAQ.bed.gz and ENCFF116DCE.bigWig), H3K27me3(ENCFF336CRJ.bigWig), H3K9me3 (ENCFF711NWS.bigWig), ELF1 (ENCFF688GOC.bigWig), GATA1 (ENCFF841DLH.bed.gz and ENCFF222HZM.bigWig), MYC (ENCFF944GOL.bigWig), TAL1 (ENCFF893PQE.bed.gz and ENCFF660OIM.bigWig) and CH12.LX EP300 (ENCFF610VZA.bigWig) were downloaded from ENCODE. Foetal liver cell H3K27me3 ChIP-seq and RNA-seq data was downloaded from a public dataset (Alvarez-Dominguez et al., 2014 [21]) and converted to mm10. ChIP-seq data for asynchronous G1ER cell CTCF and RAD21 were downloaded from 4DN data portal [22,36]. MEL CTCF data from ENCODE (ENCFF502WDT.bed.gz). ATAC-seq data for G1E cells (ENCFF157AVX.bed.gz) and erythroblast (ENCFF334HOJ.bed.gz) were downloaded from ENCODE. ICGC cancer mutation data were downloaded from the ICGC data portal version – release 28.

## Code availability

The source code is available at Github (https://github.com/Chromatin-RNA/CPOXmRNA).

## Materials availability

All unique reagents and materials reported in this study will be made available upon reasonable request without restrictions.
