# Supplementary material for: Noncoding function of super enhancer derived Cpox pre-mRNA in modulating neighbouring gene expression and chromatin interactions
Source: RNA Biol. 2025 Mar 6;22(1):1–17. doi: 10.1080/15476286.2025.2475421 (PMC11913378; doi:10.1080/15476286.2025.2475421)
Supplement: Supp.docx [file KRNB_A_2475421_SM7857.docx]

**Supplemental Information**

***Supplementary Figures***

**Supplementary Figure 1**

**
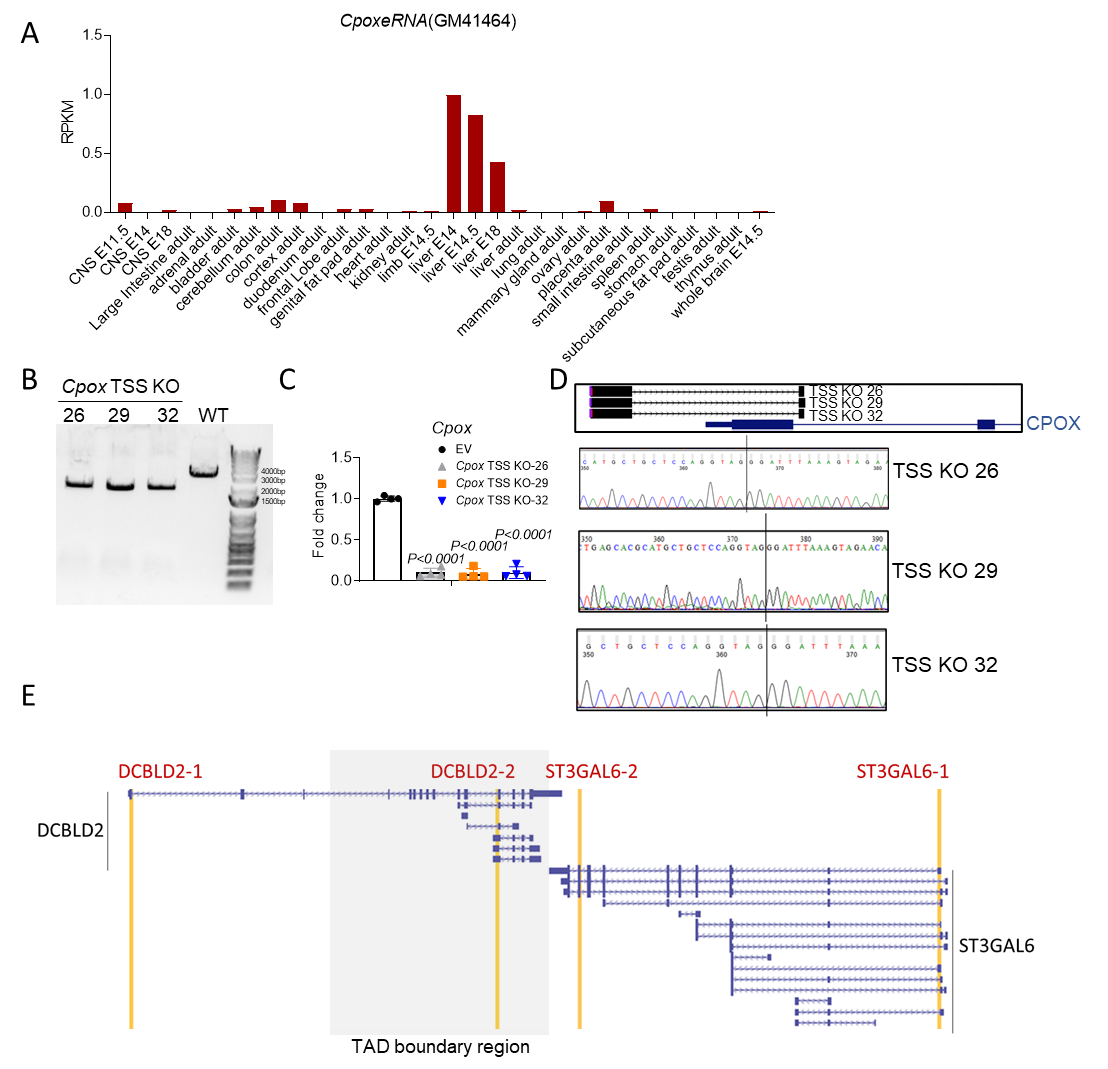
**

**Supplementary Figure 1 | *CpoxeRNA* tissue expression, *Cpox* TSS deletion, and primer location. A.** Tissue expression pattern of *CpoxeRNA* (*Gm41464*). Data from NCBI. **B.** The agarose gel image shows PCR analyses with genomic DNA from WT and *Cpox* TSS KO mutants and primer pair flanking the deletion site. **C**. *Cpox* mRNA expression level after TSS Knock out. EV, empty vector. Data are mean ± s.d.; four biological replicates. Unpaired two-tailed t-test. **D.** Sanger sequencing result for *Cpox* TSS KO, black line shows the deletion site. **E**. Location of primers for *Dcbld2* and *St3gal6* qRT-PCR. UCSC genome browser tracks show the isoforms of *Dcbld2* and *St3gal6*. Two primer pairs each used to detect *Dcbld2* and *St3gal6* are heighted in yellow. TAD boundary region is highlighted in grey.

**Supplementary Figure 2**


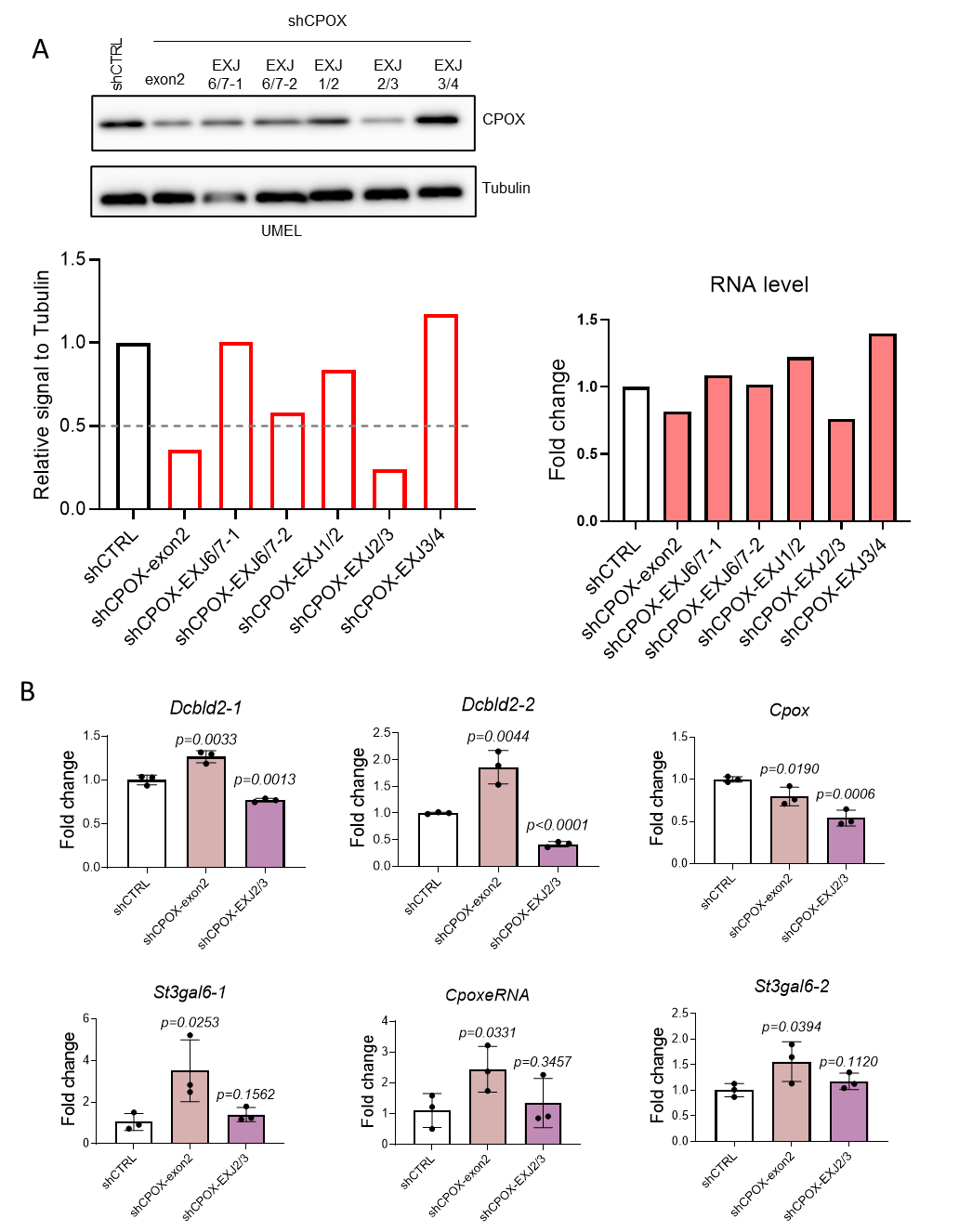


**Supplementary Figure 2 | Knock down result of shRNA targeting exon-exon junction of *Cpox*. A.** Protein and RNA level of different shRNA targeting *Cpox* exon 2 or exon-exon junctions. **B**. qRT-PCR result of target genes expression in shRNA targeting *Cpox* exon 2 or exon2 and exon3 junction. Three biological replicates. Data are mean ± s.d., unpaired one-tailed t-test.

**Supplementary Figure 3**


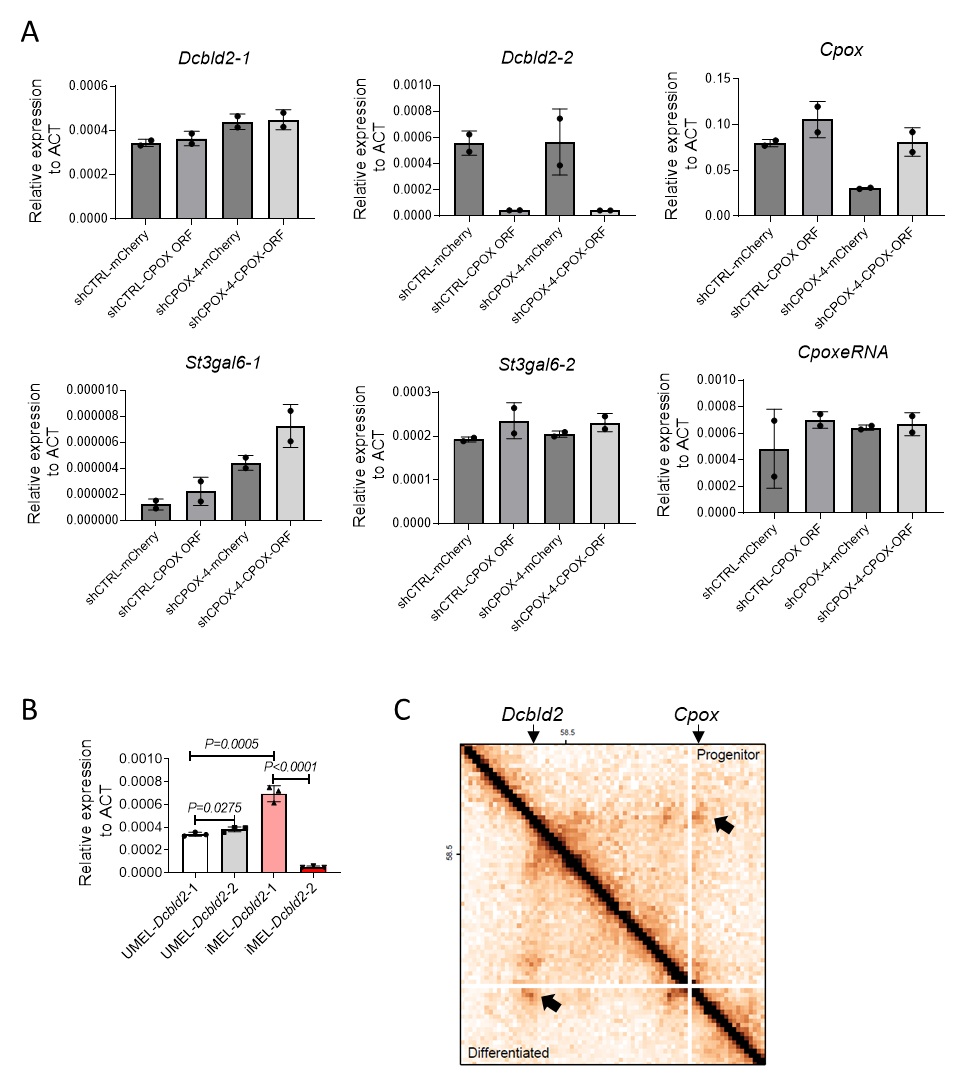


**Supplementary Figure 3 | *Cpox* ORF rescue result, Dcbld2 isoform expression level and TAD corner. A.** qRT-PCR result of target genes expression in pcDNA 3.1(+)-mCherry or pcDNA 3.1(+)-*Cpox* ORF transfected shCTRL and shCPOX-4 cells. Two biological replicates. **B.** Expression level of *Dcbld2-1* and *Dcbld2-2* in undifferentiated (UMEL) and differentiated (iMEL) MEL cells. Data are mean ± s.d.; three biological replicates. Unpaired one-tailed t-test. **C.** 5kb resolution HiC map shows the *Dcbld2-Cpox* TAD in undifferentiated and differentiated Fetal Liver cell, data from Bi et al.^38^ Black arrow shows the TAD corner loop.

**Supplementary Figure 4**


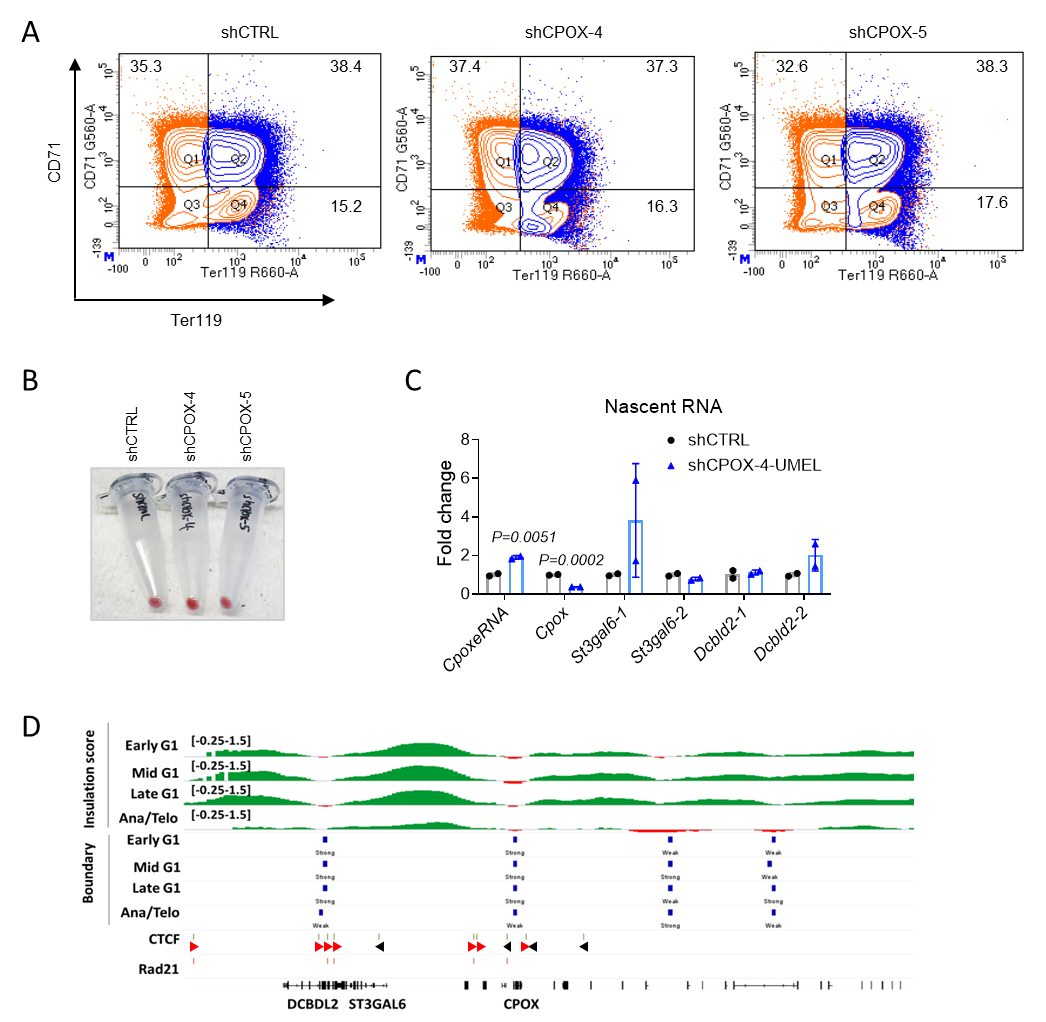


**Supplementary Figure 4 | *Cpox* knock down does not affect erythropoiesis. A.** CD71/Ter119 flow-cytometric result of differentiated shCTRL, shCPOX-4, shCPOX-5 cells. **B.** Cell pellets visualized after shCTRL, shCPOX-4, shCPOX-5 cells differentiated 5 days with 2% DMSO. **C.** qRT-PCR result shows nascent RNA expression level in shCTRL and shCPOX-4 UMEL cells. Two biological replicates. Data are mean ± s.d., unpaired one-tailed t-test. **D.** Insulation score and TAD boundary data from G1ER cell cycle in situ HiC experiment^33^ shows *Dcbld2* and *Cpox* overlap with TAD boundary. CTCF and RAD21 ChIP-seq data from asynchronous G1ER cell from the same study as G1ER cell cycle in situ HiC experiment^33^.

**Supplementary Figure 5**
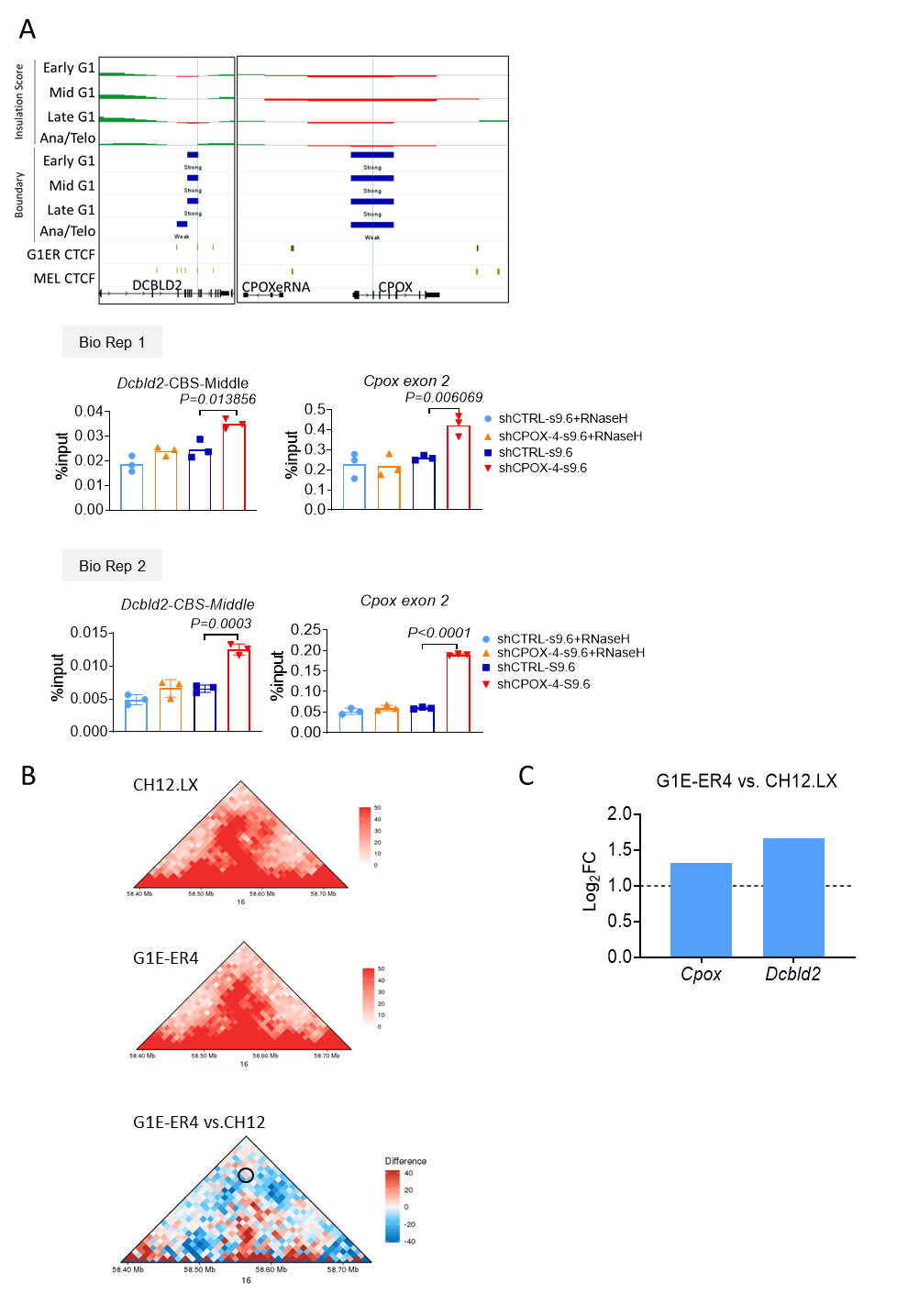


**Supplementary Figure 5** **|** **Transcription at TAD boundary correlates with TAD insulation strength. A.** Upper panel shows the insulation score, TAD boundary, and CTCF position in G1ER^33^ and MEL cells (ENCODE). Lower panel shows the DRIP-qPCR result of R loop formation at the TAD boundaries after *Cpox* mRNA knock down in UMEL cells. S9.6+RNaseH sample was the negative control sample, RNaseH was added during the pull down experiment to digest RNA-DNA hybrids. Position of primers are highlighted in the upper panel. Data are mean ± s.d.; unpaired one-tailed t-test with three technical replicates. **B.** HiC matrix shows the *Dcbld2*-*Cpox* TAD in G1ER and CH12.LX cells. Differential matrix is shown below, TAD boundary loop is highlighted with circle in the differential matrix. **C.** Fold change of *Cpox* and *Dcbld2* expression level in G1ER and CH12.LX cell. Expression data from ENCODE RNA-seq data.

**Supplementary Figure 6**


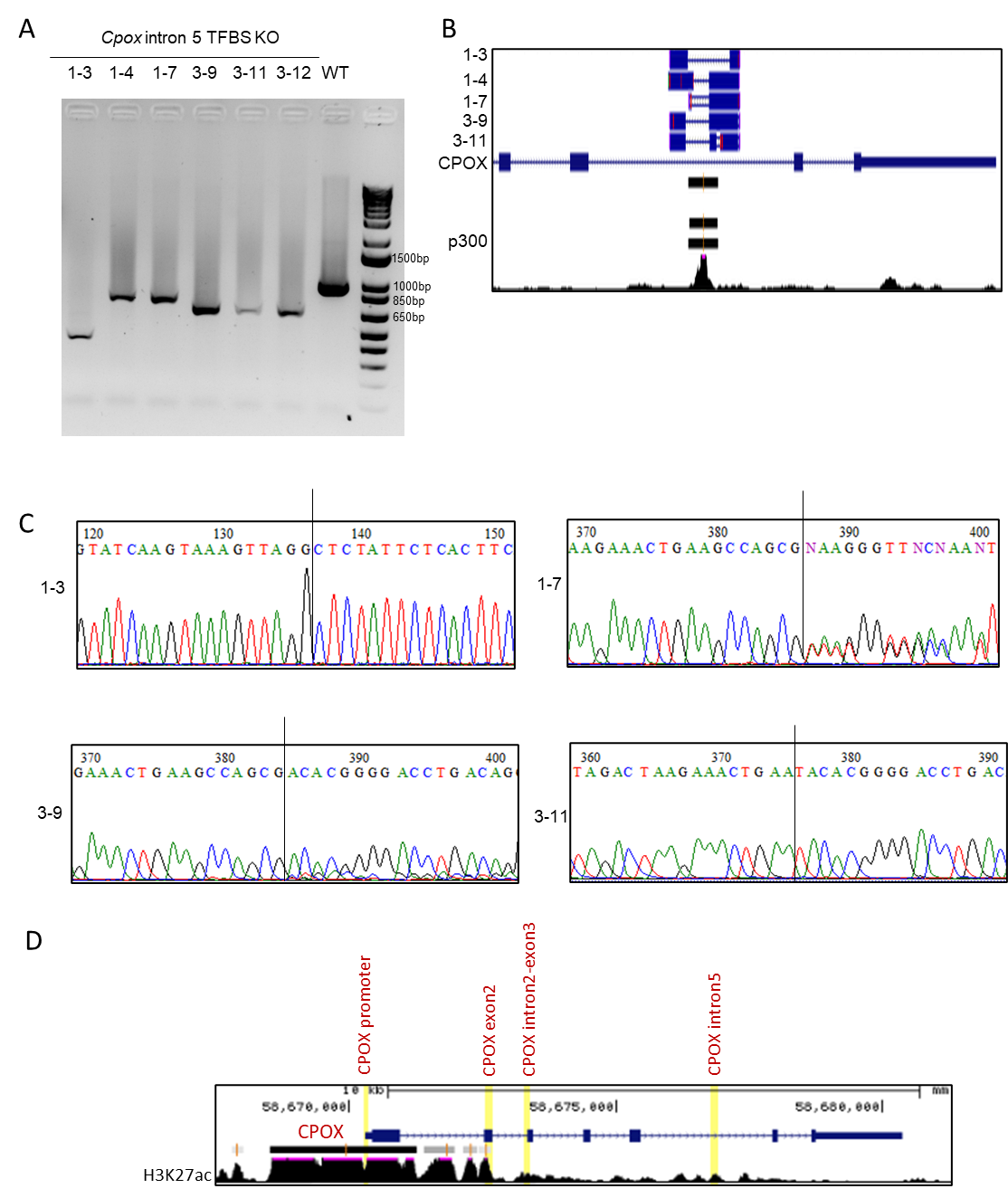


**Supplementary Figure 6 | ChIP-qPCR primer location and Sanger sequencing of genetic deletion of *Cpox* intron 5 TFBS.** **A.** The agarose gel image shows PCR analyses with genomic DNA from WT and *Cpox* intron 5 TFBS KO mutants with primer pair flanking the deletion site. **B.** *Cpox* intron 5 TFBS KO BLAST result, MEL p300 ChIP- seq data from ENCODE. **C.** Sanger sequencing data, black line shows the deletion site. **D.** Location of primers used for ChIP-qPCR in Figure 4A and B.

**Supplementary Figure 7**


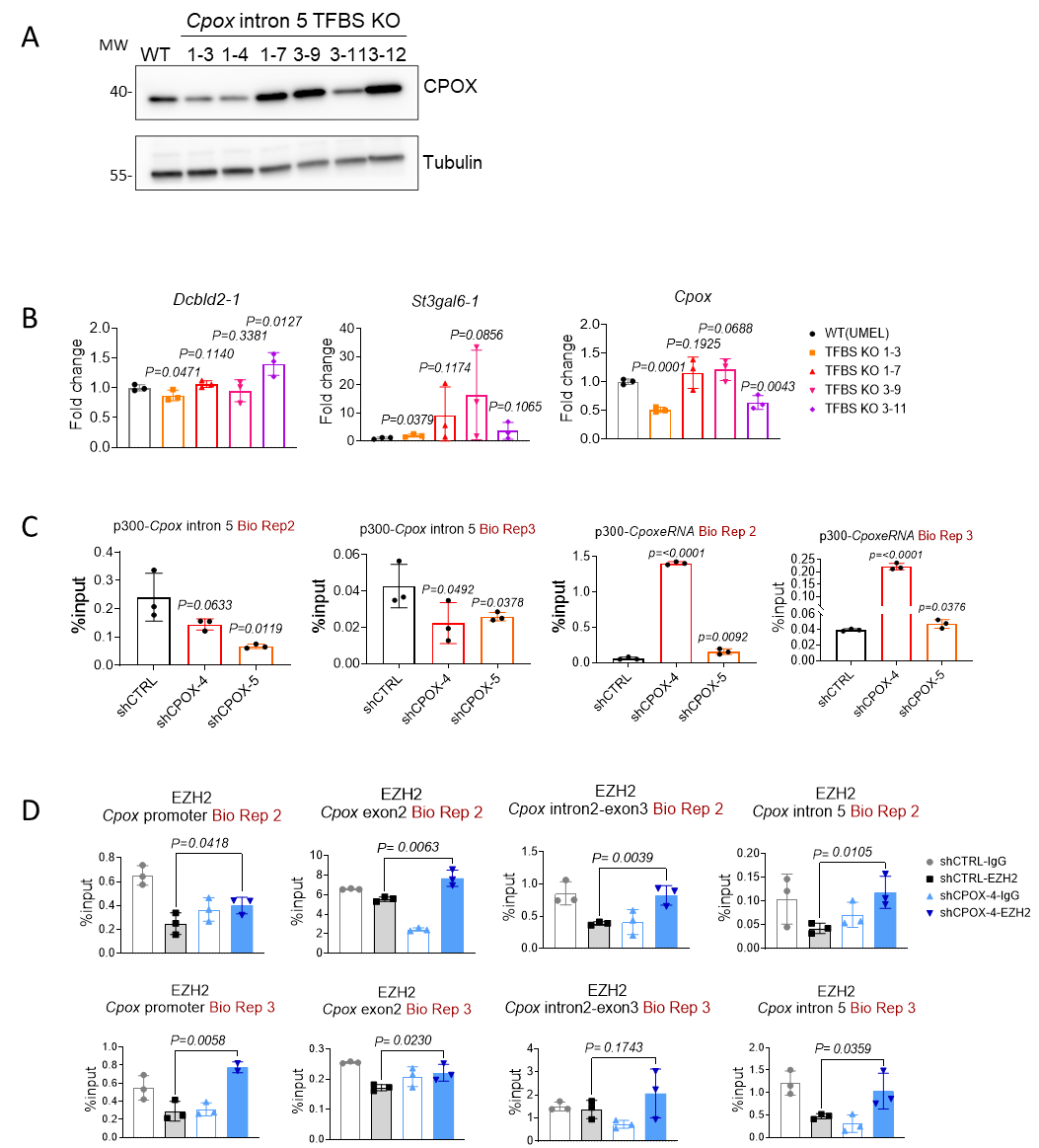


**Supplementary Figure 7 | *Cpox* intron5 TFBS deletion activates intra-TAD enhancer and TAD boundary gene *Dcbld2*. A.** Western blot result shows CPOX protein level in WT and *Cpox* intron 5 TFBS depleted cells. **B.** qRT-PCR result shows the expression of *Cpox*, *St3gal6*-1, and *Dcbld2*-1 after *Cpox* intron 5 TFBS deletion in UMEL cells. Three biological replicates. Data are mean ± s.d., unpaired one-tailed t-test. **C.** ChIP-qPCR result shows p300 binding inside TAD after *Cpox* knock down by shRNA in UMEL cells. Three independent experiments, two biological replicates shown here. Data are mean ± s.d., unpaired one-tailed t-test. **D.** ChIP-qPCR result shows EZH2 accumulation at the *Cpox* locus after *Cpox* knock down in iMEL cells. Three independent experiments, two biological replicates shown here. Data are mean ± s.d., unpaired one-tailed t-test.

**Supplementary Figure 8**
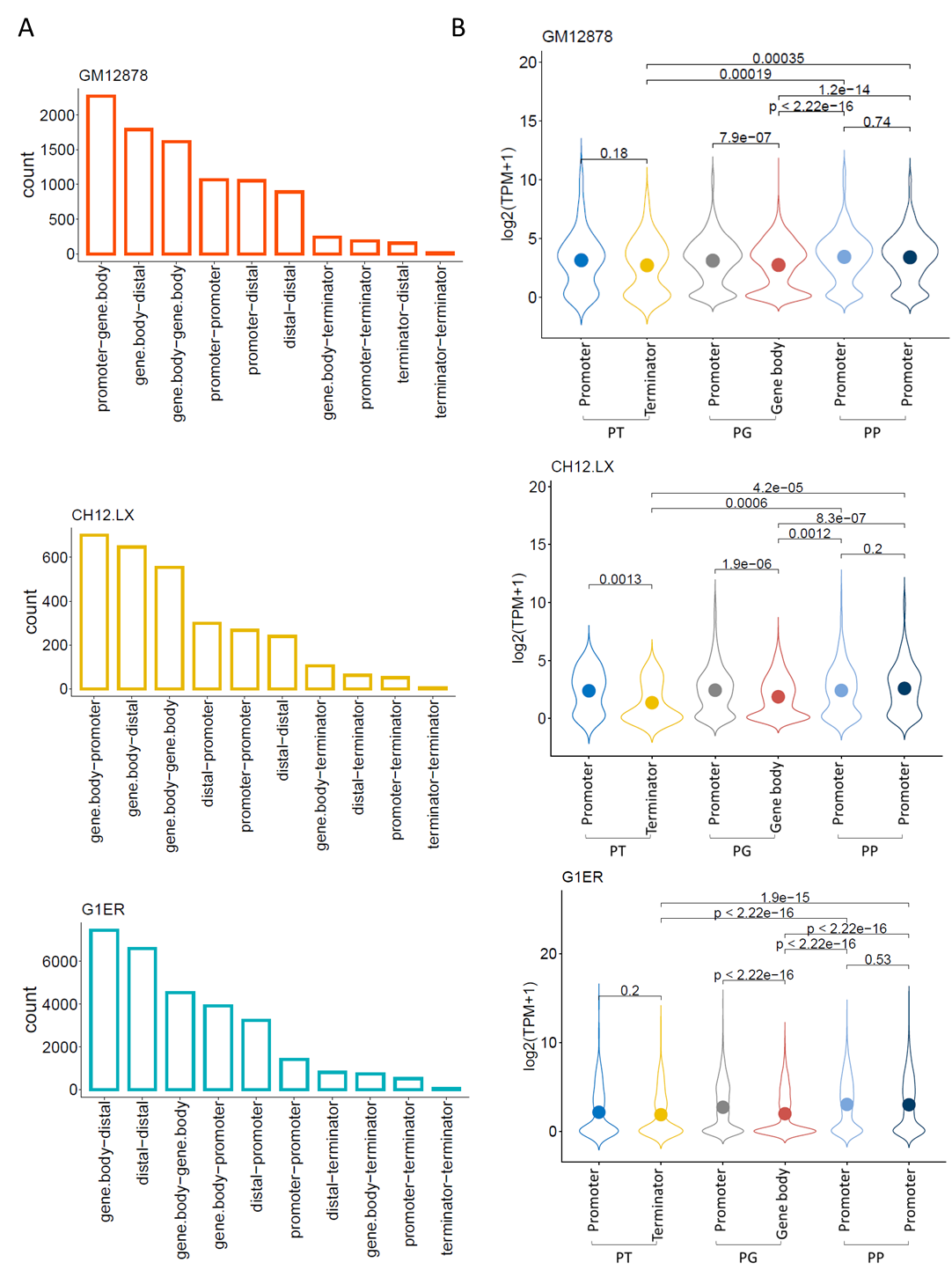


**Supplementary Figure 8** | **Distribution of TAD boundary gene interaction pattern and expression. A.** Bar plot shows the distribution of different types of interaction observed at TAD boundary in GM12878, CH12.LX, and G1ER cells. **B.** Expression level at each TAD boundary for Promoter-Terminator (PT), Promoter-Genebody (PG) and Promoter-Promoter (PP) boundary pairs in GM12878, CH12.LX, and G1ER cells. Dots inside of the violin plots show the mean value. Kruskal-Wallis test, P values ≤ 0.05 indicating a significant difference.

**Supplementary Figure 9**


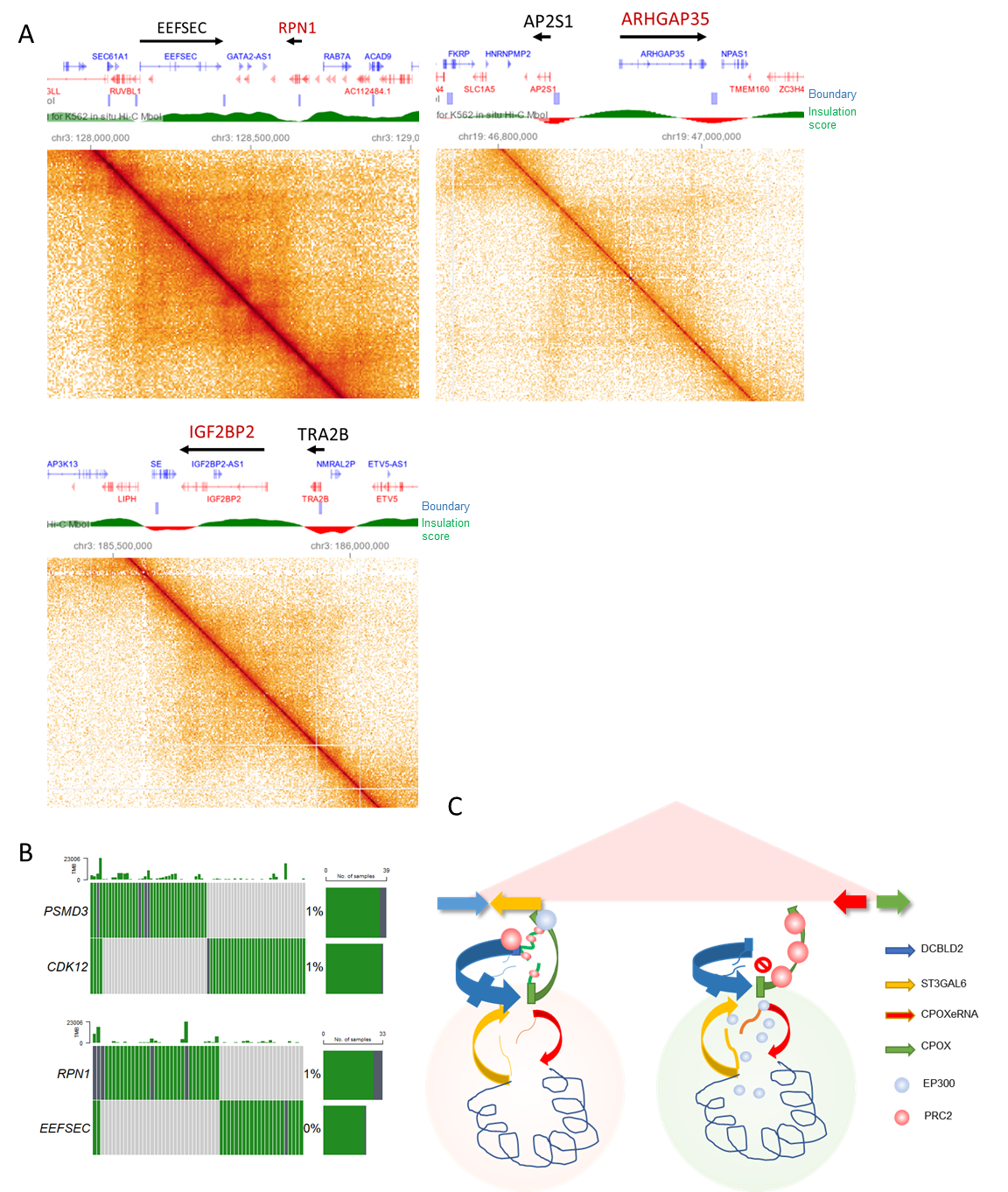


**Supplementary Figure 9** **| TAD boundary gene and TSS mutations. A.** K562 HiC maps^8,32^ of *EEFSEC*-*RPN1*, *AP2S1*-*ARHGAP35*, *IGF2BP2*-*TRA2B* pairs, insulation score and TAD boundary are shown above the HiC matrix. Names of oncogenes are in red. **B**. Oncoplots shown the landscape of ICGC mutations located in the promoters of TAD anchor non-oncogene-oncogene pairs, *PSMD3*-*CDK12* and *RPN1*-*EEFSEC*. **C.** Model of *Cpox* mRNA loss activating neighboring gene and enhancer. Left graph shows normal situation, right graph shows *Cpox* mRNA knock down situation. Colored arrows represent the genomic locus of the protein genes and enhancer. Colored curves represent the corresponding RNA transcribed. Blue dots represent p300, orange dots represent PRC2. Rectangles on genes represent the promoters.

***Supplementary Table 1***

List of primers, shRNAs, sgRNAs, and LNA ASOs used in this study. This table contains the sequence of primers, shRNAs, sgRNAs, and LNA ASOs used in this study.
